# Higher integrability for doubly nonlinear parabolic systems

**DOI:** 10.1007/s42985-022-00204-0

**Published:** 2022-10-18

**Authors:** Verena Bögelein, Frank Duzaar, Christoph Scheven

**Affiliations:** 1 Fachbereich Mathematik, Universität Salzburg, Hellbrunner Str. 34, 5020 Salzburg, Austria; 2 Department of Data Science, Universität Erlangen–Nürnberg, Cauerstrasse 11, 91058 Erlangen, Germany; 3 Fakultät für Mathematik, Universität Duisburg-Essen, Thea-Leymann-Strasse 9, 45127 Essen, Germany

**Keywords:** Doubly nonlinear parabolic equation, Higher integrability, Reverse Hölder inequality, 35B65, 35K40, 35K55

## Abstract

In this paper we establish a local higher integrability result for the spatial gradient of weak solutions to doubly nonlinear parabolic systems. The proof is based on a new intrinsic scaling that involves both the solution and its spatial gradient. It allows to compensate for the different scaling of the system in |*u*| and |*Du*|. The result covers the range of parameters $p>\frac{2n}{n+2}$ and $0<q\le 1$.

## Introduction and results

In this paper, we consider weak solutions $u:\Omega _T\rightarrow \mathbb {R}^N$ of doubly non-linear parabolic equations, resp. systems, in a space-time cylinder $\Omega _T{:}{=} \Omega \times (0,T)$, where $\Omega \subset \mathbb {R}^n$ is a bounded open domain with $n\ge 2$, $N\ge 1$, and $T>0$. The model equation is given by

1.1 $$\begin{aligned} \partial _t \big (|u|^{q-1}u\big ) -{{\,\mathrm {div}\,}}\big (|Du|^{p-2}Du\big ) = {{\,\mathrm {div}\,}}\big (|F|^{p-2}F\big ) \quad \text{ in } \Omega _T, \end{aligned}$$

with $p>1$ and $q>0$. For $q=1$ we have the parabolic *p*-Laplace, for $p=2$ we have the porous medium equation and for $q+1=p$ we recover the homogeneous doubly nonlinear equation

1.2 $$\begin{aligned} \partial _t \big (|u|^{p-2}u\big ) -{{\,\mathrm{div}\,}}\big (|Du|^{p-2}Du\big ) = {{\,\mathrm{div}\,}}\big (|F|^{p-2}F\big ) \quad \hbox {in}\; \Omega _T, \end{aligned}$$

sometimes called Trudinger’s equation. The model equation (1.1) is a special case of the general doubly nonlinear parabolic equation

1.3 $$\begin{aligned} \partial _t\big (|u|^{q-1}u\big ) -{{\,\mathrm{div}\,}}{\mathbf {A}} (x,t,u,Du) ={{\,\mathrm{div}\,}}\big (|F|^{p-2}F\big )\quad \hbox {in}\; \Omega _T, \end{aligned}$$

with a Carathéodory vector-field $\mathbf {A}:\Omega _T\times \mathbb {R}^N\times \mathbb {R}^{Nn}\rightarrow \mathbb {R}^{Nn}$ that satisfies the *p*-growth and ellipticity conditions

1.4 $$\begin{aligned} \left\{ \begin{array}{c} {\mathbf {A}}(x,t,u,\xi )\cdot \xi \ge \nu |\xi |^p\, ,\\ | {\mathbf {A}}(x,t,u,\xi )|\le L |\xi |^{p-1} \end{array} \right. \end{aligned}$$

for a.e. $(x,t)\in \Omega _T$ and any $(u,\xi )\in \mathbb {R}^N\times \mathbb {R}^{Nn}$, with constants $0<\nu \le L<\infty$. Equation (1.1) is of both physical and mathematical interest. It occurs, among other things, in filtration processes of gases or liquids in porous media. It also plays an important role in the study of non-Newtonian fluids; see [16] and [28] and references therein. It is also used to describe the dynamics of glaciers [19] and of shallow water flows [1]; see also [24] and [25]. The special case $p = 3/2$ in Trudinger’s equation (1.2) (in one space dimension) is used as a model for a friction-dominated flow in a gas network; see [2, 18]. The equation (1.1) has a different behavior when $q+1<p$ and $q+1\ge p$. In the first region perturbations propagate with finite speed and moving boundaries exist. Therefore, this region is termed the slow diffusion case. In the second range, perturbations propagate with infinite speed and extinction may occur in finite time. This range is called fast diffusion case, and Trudinger’s Eq. (1.2) represents the limiting case between the slow and fast diffusion regions.

Before stating our main result, we specify our notion of weak solution to (1.3).

### Definition 1.1

Assume that the Carathéodory vector field ${\mathbf {A}}:\Omega _T\times \mathbb {R}^N\times \mathbb {R}^{Nn}\rightarrow \mathbb {R}^{Nn}$ satisfies (1.4), and that $F\in L^p_{\mathrm{loc}} (\Omega _T,\mathbb {R}^{Nn})$. We identify a measurable map $u:\Omega _T\rightarrow \mathbb {R}^{N}$ in the class

$$\begin{aligned} u\in C \big ([0,T]; L^{q+1}(\Omega ,\mathbb {R}^N)\big ) \cap L^p\big (0,T;W^{1,p}(\Omega ,\mathbb {R}^N)\big ) \end{aligned}$$

as a *weak solution* to the doubly non-linear parabolic Eq. (1.3) if and only if the identity

1.5 $$\begin{aligned} \iint _{\Omega _T}\big [|u|^{q-1}u\cdot \varphi _t - {\mathbf {A}}(x,t,u,Du)\cdot D\varphi \big ]\mathrm {d}x\mathrm {d}t= \iint _{\Omega _T} |F|^{p-2}F\cdot D\varphi \,\mathrm {d}x\mathrm {d}t\end{aligned}$$

holds, for any testing function $\varphi \in C_0^\infty (\Omega _T,\mathbb {R}^N)$. $\square$

The purpose of this paper is to establish a local higher integrability result for the spatial gradient of weak solutions to doubly non-linear parabolic equations and systems of the type (1.3).

### Theorem 1.2

Let $p>\frac{2n}{n+2}$, $0<q\le 1$, $\sigma >p$, and $F\in L^\sigma _{\mathrm{loc}}(\Omega _T,\mathbb {R}^N)$. Then, there exist $\varepsilon _o=\varepsilon _o(n,p,q,\nu ,L)\in (0,1]$ and $c=c(n,p,q,\nu ,L)\ge 1$ such that whenever *u* is a weak solution of (1.3) in the sense of Definition 1.1, then there holds

1.6 $$\begin{aligned} D u \in L^{p(1+\varepsilon _1)}_{\mathrm{loc}}\big (\Omega _T,\mathbb {R}^{Nn}\big ), \end{aligned}$$

where $\varepsilon _1{:}{=}\min \{\varepsilon _o,\frac{\sigma }{p}-1\}$. Moreover, for every $\varepsilon \in (0,\varepsilon _1]$ and every cylinder $Q_{\varrho }{:}{=}B_\varrho (x_o)\times (t_o-\varrho ^{1+q}, t_o+\varrho ^{1+q})\Subset \Omega _T$, we have the quantitative local higher integrability estimate1.7with $p^\sharp {:}{=}\max \{p,q+1\}$ and the scaling deficit

1.8 $$\begin{aligned} d{:}{=} \left\{ \begin{array}{cl} \frac{p}{q+1},&{}\hbox {for }p\ge q+1,\\ \frac{p(q+1)}{p(q+1)+n(p-q-1)},&{}\hbox {for }\frac{2n}{n+2}<p<q+1. \end{array} \right. \end{aligned}$$

**Fig. 1:**
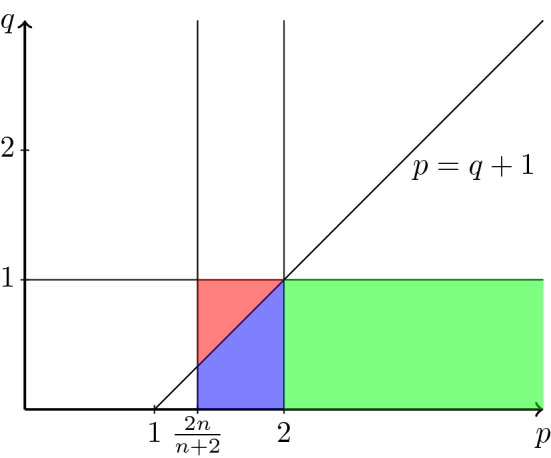
Range of *p* and *q*

Theorem 1.2 ensures in the range $(p,q)\in \big (\frac{2n}{n+2},\infty )\times (0,1]$ that weak solutions of (1.3) belong to a slightly better Sobolev space than the natural energy space and therefore obey a self-improving property of integrability. The improvement in integrability of the spatial gradient, as stated in (1.6), is the direct consequence of the quantitative reverse Hölder type estimate (1.7). The range of (*p*, *q*) covered by Theorem  1.2 is composed of three parts that are illustrated in the diagram in Fig. 1 below. The part that lies above the line $q=p-1$ (the red triangle) belongs to the fast diffusion range, while the parts below the line (the green and blue region) are contained in the slow diffusion range. The green and the blue region differ in the properties of the diffusion part of the differential equation (1.3), which becomes degenerate for $p\ge 2$ (the green region) and singular for $p<2$ (the blue region). By this we mean that the modulus of ellipticity degenerates for small values of |*Du*| in the green region, while it becomes singular in the blue region. Because of the differences described above, each of the three regions requires slightly different techniques. The common feature of the three colored regions is that we can work with cylinders as in (2.2) that are scaled in time, while the cases with $q>1$ require a scaling in the spatial directions, as it has been used for the singular porous medium equation in [7, 12]. This is the reason why we restrict ourselves to the cases in which $0<q\le 1$.

The lower bound $p>\frac{2n}{n+2}$ in Theorem  1.2 already emerges in the case of the parabolic *p*-Laplace system; cf. [17]. It is needed in the proof of the Sobolev-Poincaré inequality, see Lemma 4.4. Theorem 1.2 contains, among others, as limiting cases the parabolic *p*-Laplace system $q=1$, $p> \frac{2n}{n+2}$, which was originally treated in [17], the slow diffusion range for the porous medium equation $0<q\le 1$, $p=2$, cf. [6, 11], and Trudinger’s equation, i.e. the case when $q=p-1$, in the range $\frac{2n}{n+2}<p\le 2$; cf. [5]. We should point out that in these special cases inequality (1.7) reproduces the reverse Hölder type inequalities established in [5, 6, 17].

The key to the proof of our main result is a suitable intrinsic geometry, a concept which was originally introduced by DiBenedetto and Friedman [9]; see also the monographs [10, 27]. Meanwhile, variants of this idea have been successfully used to prove higher integrability for the parabolic *p*-Laplace system [17] and more recently for porous medium type equations [11, 12], for porous medium type systems [6, 7], and for Trudinger’s equation [5]. Our idea is to consider space-time cylinders $Q_{\varrho ,s}(z_o){:}{=}B_\varrho (x_o)\times (t_o-s,t_o+s)$, with $z_o=(x_o,t_o)$, such that the quotient $\frac{s}{\varrho ^{1+q}}$ satisfies

$$\begin{aligned} \frac{s}{\varrho ^{1+q}} = \lambda ^{2-p}\theta ^{q-1}, \end{aligned}$$

with1.9Thus, the scaling in time direction depends on both, the solution and its spatial gradient. It allows us to compensate for the different scaling of |*u*| and |*Du*| in the system. To our knowledge, this is the first time such a geometry is used in the context of gradient estimates for doubly nonlinear equations. We note that a related construction of cylinders, which also depend on two parameters, was used in a different context in [26]. On the cylinders described above, we are able to prove Sobolev–Poincaré and reverse Hölder type inequalities. The construction of the cylinders is quite complex, since the domain of integration on the right-hand side of (1.9) also depends on the parameters $\lambda$ and $\theta$. This is achieved in the final part of the paper. For any $\lambda$ and any radius $\varrho$ we find $\theta =\theta (\lambda , \varrho )$ such that (1.9)$_2$ is satisfied. However, since $\theta$ does not depend monotonically on $\varrho$ we have to modify our choice at the cost that the identity in (1.9)$_2$ has to be replaced by ’$\ge$’. Such cylinders are called $\theta$-sub-intrinsic. In the course of the construction we modify an argument from [11]; see also [5–7, 23]. Next, we fix a parameter $\lambda$ and consider the super-level set $\{|Du|>\lambda \}$. For any point in this set, we find by a stopping time argument a radius $\varrho$, which in turn fixes $\theta (\lambda ,\varrho )$, such that the associated cylinder satisfies (1.9)$_1$. By construction the cylinder is also $\theta$-sub-intrinsic. On these cylinders we can apply the previously proved reverse Hölder inequality. However, some complications occur, since they are only $\theta$-sub-intrinsic. The final part of the proof is quite standard. Once the reverse Hölder inequalities are established, we conclude gradient estimates on super-level sets. Integration with respect to the levels $\lambda$ then yields the quantitative higher integrability estimate.

At this point, a few words to place our result in the history of the problem of higher integrability are in order. In the stationary elliptic case, the higher integrability was first observed by Elcrat & Meyers [20], see also the monographs [13, Chapter 11, Theorem 1.2] and [15, Sect. 6.5] and the references therein. The first higher integrability result for parabolic systems goes back to Giaquinta & Struwe [14, Theorem 2.1]. For parabolic systems with *p*-growth whose main prototype is the parabolic *p*-Laplace system, the higher integrability of the gradient of weak solutions was established by Kinnunen & Lewis [17] in the range $p>\frac{2n}{n+2}$. This lower bound is natural and also appears in other contexts in the regularity theory of parabolic *p*-Laplace systems; cf. the monograph [10]. In the meantime, the result has been generalized in several directions, such as global results, higher order parabolic systems with *p*-growth, and parabolic systems with *p*(*x*, *t*)-growth; cf. [3, 4, 8, 21]. The corresponding problem for the porous medium equation, i.e. (1.1) with $p=2$, proved to be more complicated and remained open for a long time, even in the scalar case for non-negative solutions. In addition to the obvious anisotropic behavior of the equation with respect to scalar multiplication of solutions, it is also not possible to add constants to a solution without losing the property of being a solution. This difficulty has recently been overcome by Gianazza & Schwarzacher [11] who proved in the slow diffusion range $0<q<1$ that non-negative weak solutions of porous medium type equations admit the self-improving property of higher integrability of the gradient. The main novelty in their proof is the use of a new intrinsic scaling. Instead of scaling cylinders with respect to |*Du*| as in the case of the parabolic *p*-Laplace (cf. [10] and the references therein), they work with cylinders which are intrinsically scaled with respect to |*u*|. The proof, however, uses the method of expansion of positivity and therefore can not be extended to signed solutions, to the case of porous medium type systems, and to the fast diffusion range. A simpler and more flexible proof that does not rely on the expansion of positivity and that covers both signed solutions and vector-valued solutions, can be found in [6]. The singular case of the porous medium equation has been treated independently in [12] for non-negative solutions and in [7] for vector-valued solutions. Next, in [5] the higher integrability is established for Trudinger’s equation. In this equation aspects of both the porous medium equation and the parabolic *p*-Laplace equation play a role. Therefore the intrinsic scaling has to take into account the degeneracy of the system both with respect to the gradient variable and with respect to the solution itself. In [5] the higher integrability is established for exponents *p* in the somewhat unexpected range $\max \{\frac{2n}{n+2},1\}<p<\frac{2n}{(n-2)_+}$. The lower bound also appears for the parabolic *p*-Laplace system [17], while the upper bound corresponds exactly to the lower bound for the porous medium equation in the fast diffusion range. In the scalar case of non-negative solutions of Trudinger’s equation with $F=0$, the upper bound could be eliminated in [22]. However, the techniques used in [22] are strictly limited to the scalar case.

## Preliminaries

### Notations

Throughout the paper, we use space-time cylinders of the form

$$\begin{aligned} Q_\varrho ^{(\lambda ,\theta )}(z_o){:}{=} B_\varrho (x_o)\times \Lambda _\varrho ^{(\lambda ,\theta )}(t_o), \end{aligned}$$

with center $z_o=(x_o,t_o)\in \mathbb {R}^n\times \mathbb {R}$, radius $\varrho >0$ and scaling parameters $\lambda ,\theta >0$. Here

$$\begin{aligned} \Lambda _\varrho ^{(\lambda ,\theta )}(t_o){:}{=} \big (t_o-\tau _\varrho ^{(\lambda ,\theta )}, t_o+\tau _\varrho ^{(\lambda ,\theta )}\big ), \end{aligned}$$

where

2.1 $$\begin{aligned} \tau _\varrho ^{(\lambda ,\theta )} {:}{=} \tau ^{(\lambda ,\theta )}\varrho ^{1+q}, \quad \hbox {with}\qquad \tau ^{(\lambda ,\theta )}{:}{=}\theta ^{q-1}\lambda ^{2-p}. \end{aligned}$$

For the case that $\theta =1=\lambda$, we simply omit the scaling parameters in our notation and instead of $Q_\varrho ^{(1,1)}(z_o)$ write

2.2 $$\begin{aligned} Q_\varrho (z_o){:}{=}B_\varrho (x_o)\times (t_o-\varrho ^{1+q},t_o+\varrho ^{1+q}). \end{aligned}$$

For a mapping $u\in L^1\big (0,T;L^1(\Omega ,\mathbb {R}^N)\big )$ and a given measurable set $A\subset \Omega$ with positive Lebesgue measure the slice-wise mean $(u)_{A}:(0,T)\rightarrow \mathbb {R}^N$ of *u* on *A* is defined by2.3Note that if $u\in C^0\big ([0,T];L^{1+q}(\Omega ,\mathbb {R}^N)\big )$ the slicewise means are defined for any $t\in [0,T]$. If the set *A* is a ball $B_\varrho (x_o)$, then we abbreviate $(u)_{x_o;\varrho }(t){:}{=}(u)_{B_\varrho (x_o)}(t)$. Similarly, for a given measurable set $E\subset \Omega \times (0,T)$ of positive Lebesgue measure the mean value $(u)_{E}\in \mathbb {R}^N$ of *u* on *E* is defined byIf $E= Q_\varrho ^{(\lambda ,\theta )}(z_o)$, we use the short hand notation $(u)^{(\lambda ,\theta )}_{z_o;\varrho }{:}{=}(u)_{Q_\varrho ^{(\lambda ,\theta )}(z_o)}$.

For a power of a vector $u\in \mathbb {R}^N$, we use the notationfor $\alpha >0$, which we interpret as  in the case $u=0$. Finally, we define the boundary termfor any $u,a\in \mathbb {R}^N$.

### Auxiliary material

In order to “re-absorb” certain quatities, we will use the following iteration lemma, which can be retrieved by a simple change of variable from [15, Lemma 6.1].

#### Lemma 2.1

Let $0<\vartheta <1$, $A,C\ge 0$ and $\alpha ,\beta > 0$. Then there exists a universal constant $c = c(\alpha ,\beta ,\vartheta )$ such that there holds: For any non-negative bounded function satisfying

$$\begin{aligned} \phi (t) \le \vartheta \phi (s) + A (s^\alpha -t^\alpha )^{-\beta } + C \qquad \text {for all} \,\, 0<r\le t<s\le \varrho , \end{aligned}$$

we have

$$\begin{aligned} \phi (r) \le c\, \big [A (\varrho ^\alpha - r^\alpha )^{-\beta } + C\big ]. \end{aligned}$$

The next lemma can be verified using the arguments from [15, Lemma 8.3].

#### Lemma 2.2

For any $\alpha >0$, there exists a constant $c=c(\alpha )$ such that, for all $a,b\in \mathbb {R}^N$, $N\in {\mathbb {N}}$, we have

As an easy consequence of the preceding lemma, we obtain

#### Lemma 2.3

For any $\alpha \ge 1$, there exists a constant $c=c(\alpha )$ such that, for all $a,b\in \mathbb {R}^N$, $N\in {\mathbb {N}}$, we have

The following estimate, which is known as the quasi-minimality of the mean value, can be retrieved from [5, Lemma 3.5].

#### Lemma 2.4

Let $p\ge 1$ and $\alpha \ge \frac{1}{p}$. Then, there exists a constant $c=c(\alpha ,p)$ such that whenever $A\subseteq B\subset \mathbb {R}^k$, $k\in {\mathbb {N}}$, are two bounded domains of positive measure, then for any function $u \in L^{\alpha p}(B,\mathbb {R}^N)$ and any constant $a\in \mathbb {R}^N$, we have

The proof of the following lemma can be found in [6, Lemma 2.3 (i)] for the case $0<q\le 1$ and in [5, Lemma 3.4] for parameters $q>1$.

#### Lemma 2.5

For any $q>0$ there exists a constant $c=c(q)$ such that for any $u,v\in \mathbb {R}^N$, $N\in {\mathbb {N}}$, the following estimates hold true:

Finally, we recall Gagliardo-Nirenberg’s inequality, which can be formulated in the following way.

#### Lemma 2.6

Let $1\le p,q,r<\infty$ and $\theta \in (0,1)$ such that $- \frac{n}{p} \le \theta (1 - \frac{n}{q} ) - ( 1- \theta ) \frac{n}{r}$. Then there exists a constant $c=c(n,p)$ such that for any ball $B_\varrho (x_o)\subset \mathbb {R}^n$ with $\varrho >0$ and any function $u \in W^{1,q}(B_\varrho (x_o))$, we have

## Energy bounds

In this section we provide a Caccioppoli inequality and a gluing lemma for solutions to the doubly non-linear system. Thereby, we include the whole range $p>1$ and $q>0$, since the proofs of these lemmas do not differ when *p* or *q* vary. We start with the Caccioppoli inequality.

### Lemma 3.1

Let $p>1$, $q>0$ and u be a weak solution to (1.3) in $\Omega _T$ in the sense of Definition 1.1. Then, on each cylinder $Q_{\varrho }^{(\lambda ,\theta )}(z_o)\subseteq \Omega _T$ with $0<\varrho \le 1$ and $\lambda ,\theta >0$, and for all $r\in [\varrho /2,\varrho )$ and all $a\in \mathbb {R}^N$ the following energy estimateholds true for a universal constant $c=c(p,q,\nu ,L)$.

### Proof

For $v\in L^1(\Omega _T,\mathbb {R}^N)$, we define a mollification in time by

$$\begin{aligned} \llbracket v \rrbracket _h(x,t){:}{=} \tfrac{1}{h} \int _0^t \mathrm e^{\frac{s-t}{h}} v(x,s) \, \mathrm {d}s. \end{aligned}$$

From the weak form (1.5) of the differential equation we deduce the mollified version3.1for any $\varphi \in L^p(0,T;W^{1,p}_0(\Omega ,\mathbb {R}^N))\cap L^{q+1}(\Omega _T,\mathbb {R}^N)$. Throughout the rest of the proof we omit the reference to the center $z_o=(x_o,t_o)$ in our notation. Let $\eta \in C^1_0(B_\varrho ,[0,1])$ be a cut-off function with $\eta \equiv 1$ in $B_r$ and $|D\eta |\le \frac{2}{\varrho -r}$, and define $\zeta \in W^{1,\infty } (\Lambda ^{(\lambda ,\theta )}_\varrho (t_o),[0,1])$ by

$$\begin{aligned} \zeta (t) {:}{=} \left\{ \begin{array}{cl} {\displaystyle \frac{\frac{t-t_o}{\tau ^{(\lambda ,\theta )}} + \varrho ^{1+q}}{\varrho ^{1+q}-r^{1+q}} },&{} \hbox {for} \,\,t\in \big (t_o-\tau _\varrho ^{(\lambda ,\theta )}, t_o-\tau _r^{(\lambda ,\theta )}\big ),\\ 1, &{} \hbox {for}\,\, t\in \big [ t_o- \tau _r^{(\lambda ,\theta )}, t_o+ \tau _\varrho ^{(\lambda ,\theta )}\big ). \end{array} \right. \end{aligned}$$

Furthermore, for $\varepsilon >0$ small enough and $t_1 \in \Lambda ^{(\lambda ,\theta )}_r(t_o)$ we define the function $\psi _\varepsilon \in W^{1,\infty } (\Lambda ^{(\theta )}_\varrho (t_o),[0,1])$ by

$$\begin{aligned} \psi _\varepsilon (t) {:}{=} \left\{ \begin{array}{cl} 1 &{} \hbox {for}\,\, t\in \big (t_o-\tau _\varrho ^{(\lambda ,\theta )}, t_1\big ], \\ 1-\frac{1}{\varepsilon } (t-t_1) &{} \hbox {for}\,\, t\in (t_1, t_1+\varepsilon ), \\ 0 &{} \hbox {for}\,\, t\in \big [t_1+\varepsilon , t_o+\tau _\varrho ^{(\lambda ,\theta )}\big ). \end{array} \right. \end{aligned}$$

With these choices, (3.1) can be tested with

$$\begin{aligned} \varphi (x,t){:}{=} \eta ^p(x) \psi _\varepsilon (t) \zeta (t)\big (u(x,t)-a\big ). \end{aligned}$$

In the following, let . For the integral containing the time derivative we getwhere we used the fact , which follows from the elementary identitySince $\llbracket u\rrbracket _h\rightarrow u$ in $L^{q+1}_\mathrm{loc}(\Omega _T)$, we can pass to the limit $h\downarrow 0$ in the integral on the right-hand side. We obtainIn the left-hand side of the preceding inequality we pass to the limit $\varepsilon \downarrow 0$. For the term $\mathrm {I}_{\varepsilon }$ we obtain for any $t_1 \in \Lambda _{r}^{(\lambda ,\theta )}(t_o)$ that

$$\begin{aligned} \lim _{\varepsilon \downarrow 0}\mathrm {I}_\varepsilon = \int _{B_\varrho } \eta ^p {\mathfrak {b}}[u(t_1), a] \,\mathrm {d}x, \end{aligned}$$

whereas the term $\mathrm {II}_\varepsilon$ can be estimated in the following way (observe that the boundary term is non-negative)

$$\begin{aligned} |\mathrm {II}_\varepsilon | \le \iint _{Q_{\varrho }^{(\lambda ,\theta )}} \zeta ' {\mathfrak {b}}[u,a] \,\mathrm {d}x\mathrm {d}t\le \iint _{Q_\varrho ^{(\lambda , \theta )}} \frac{{\mathfrak {b}}[u,a]}{ \tau _\varrho ^{(\lambda ,\theta )}-\tau _r^{(\lambda ,\theta )}} \,\mathrm {d}x\mathrm {d}t. \end{aligned}$$

Next, we consider the diffusion term. After passing to the limit $h\downarrow 0$, we use the ellipticity and growth assumption (1.4) for the vector-field ${\mathbf {A}}$, and later on Young’s inequality. In this way, we obtain

$$\begin{aligned} \iint _{Q_\varrho ^{(\lambda , \theta )}}&{\mathbf {A}}(x,t,u,Du) \cdot D \varphi \, \mathrm {d}x\mathrm {d}t\\&= \iint _{Q_\varrho ^{(\lambda , \theta )}} {\mathbf {A}} (x,t,u,Du) \cdot \big [ \eta ^p \zeta \psi _\varepsilon Du + p \eta ^{p-1} \zeta \psi _\varepsilon (u-a)\otimes D\eta \big ] \,\mathrm {d}x\mathrm {d}t\\&\ge \nu \iint _{Q_\varrho ^{(\lambda , \theta )}} \eta ^p \zeta \psi _\varepsilon |Du|^p \mathrm {d}x\mathrm {d}t- Lp\iint _{Q_\varrho ^{(\lambda , \theta )}} \eta ^{p-1} |D\eta | \zeta \psi _\varepsilon |u-a| |Du|^{p-1} \,\mathrm {d}x\mathrm {d}t\\&\ge \tfrac{\nu }{2} \iint _{Q_\varrho ^{(\lambda , \theta )}} \eta ^p \zeta \psi _\varepsilon |Du|^p \mathrm {d}x\mathrm {d}t- c\iint _{Q_\varrho ^{(\lambda , \theta )}}|D\eta |^p |u-a|^p \,\mathrm {d}x\mathrm {d}t\\&\ge \tfrac{\nu }{2} \iint _{Q_\varrho ^{(\lambda , \theta )}} \eta ^p \zeta \psi _\varepsilon |Du|^p \mathrm {d}x\mathrm {d}t- c \iint _{Q_\varrho ^{(\lambda , \theta )}}\frac{|u-a|^p}{(\varrho -r)^p} \,\mathrm {d}x\mathrm {d}t, \end{aligned}$$

with a constant $c=c(p,\nu ,L)$. Finally, we consider the right-hand side term, i.e. the term involving the inhomogeneity *F*. This term can be estimated with Young’s inequality. We conclude that

$$\begin{aligned} \bigg |\iint _{Q_\varrho ^{(\lambda , \theta )}}&|F|^{p-2}F\cdot D\varphi \,\mathrm {d}x\mathrm {d}t\bigg |\\&\le \iint _{Q_\varrho ^{(\lambda , \theta )}}\zeta \psi _\varepsilon |F|^{p-1}\Big [ p\eta ^{p-1}|D\eta | |u-a| + \eta ^p|Du|\Big ]\,\mathrm {d}x\mathrm {d}t\\&\le \tfrac{\nu }{4}\iint _{Q_\varrho ^{(\lambda , \theta )}}\zeta \psi _\varepsilon |Du|^p \,\mathrm {d}x\mathrm {d}t+ c \iint _{Q_\varrho ^{(\lambda , \theta )}} \bigg [\frac{|u-a|^p}{(\varrho -r)^p} + |F|^p\bigg ] \,\mathrm {d}x\mathrm {d}t, \end{aligned}$$

with a constant $c=c(p,\nu )$. By standard properties of the mollification, we obtainWe combine these inequalities and pass to the limit $\varepsilon \downarrow 0$. In this way we get that for almost every $t_1 \in \Lambda _r^{(\theta )}(t_o)$ there holds

$$\begin{aligned} \int _{B_r}&{\mathfrak {b}} [u(t_1),a] \,\mathrm {d}x+ \int _{t_o-\tau _r^{(\lambda ,\theta )}}^{t_1}\int _{B_r} |Du|^p \,\mathrm {d}x\mathrm {d}t\\&\le c \iint _{Q_\varrho ^{(\lambda , \theta )}} \bigg [\frac{|u-a|^p}{(\varrho -r)^p} + \frac{{\mathfrak {b}}[u,a]}{ \tau _\varrho ^{(\lambda ,\theta )}-\tau _r^{(\lambda ,\theta )}} + |F|^p \bigg ] \,\mathrm {d}x\mathrm {d}t. \end{aligned}$$

Here we pass to the supremum over $t_1\in \Lambda _\varrho ^{(\lambda , \theta )}(t_o)$ in the first term on the left-hand side and then let $t_1\uparrow t_o$ in the second one. Finally, we take mean values on both sides and recall the definition of $\tau _\varrho ^{(\lambda ,\theta )}$. In view of Lemma 2.5, this yields the claimed energy estimate with a constant $c=c(p,q,\nu ,L)$. $\square$

Now, we turn out attention to the gluing lemma.

### Lemma 3.2

Let $p>1$, $q>0$ and *u* be a weak solution to (1.3) in $\Omega _T$ in the sense of Definition 1.1, where the vector-field ${\mathbf {A}}$ fulfills the growth and ellipticity assumptions (1.4). Then, on any cylinder $Q_{\varrho }^{(\lambda ,\theta )}(z_o)\subseteq \Omega _T$ with $0<\varrho \le 1$ and $\lambda ,\theta >0$ there exists ${\hat{\varrho } \in } [\frac{\varrho }{2},\varrho ]$ such that for all $t_1,t_2\in \Lambda _\varrho ^{(\lambda ,\theta )}(t_o)$ there holds

### Proof

Let $t_1,t_2\in \Lambda _\varrho ^{(\lambda ,\theta )}(t_o)$ with $t_1<t_2$ and assume that $r \in [\frac{\varrho }{2}, \varrho ]$. For $\delta >0$ and $0<\varepsilon \ll 1$, we define $\xi _\varepsilon \in C^\infty _0(t_1-\varepsilon ,t_2+\varepsilon )$ by

$$\begin{aligned} \xi _\varepsilon (t) {:}{=} \left\{ \begin{array}{cl} 0 ,&{} \hbox {for}\,\,\, t_o-\tau _\varrho ^{(\lambda ,\theta )}\le t\le t_1-\varepsilon ,\\ \frac{t-t_1+\varepsilon }{\varepsilon }, &{} \hbox {for} \,\,\,t_1-\varepsilon< t< t_1,\\ 1 ,&{} \hbox {for}\,\,\, t_1\le t\le t_2,\\ \frac{t_2+\varepsilon -t}{\varepsilon }, &{} \hbox {for}\,\,\, t_2< t< t_2+\varepsilon ,\\ 0 ,&{} \hbox {for}\,\,\, t_2+\varepsilon \le t\le t_o+\tau _\varrho ^{(\lambda ,\theta )} \end{array} \right. \end{aligned}$$

and a radial function $\Psi _\delta \in W^{1,\infty }_0(B_{r+\delta }(x_o))$ by $\Psi _\delta (x){:}{=}\psi _\delta (|x-x_o|)$, where

$$\begin{aligned} \psi _\delta (s){:}{=} \left\{ \begin{array}{cl} 1 ,&{} \hbox {for}\,\,\, 0\le s\le r,\\ \frac{r+\delta -s}{\delta }, &{} \hbox {for}\,\,\, r< s< r+\delta ,\\ 0 ,&{} \hbox {for}\,\,\,r+\delta \le s\le \varrho . \end{array} \right. \end{aligned}$$

For fixed $i\in \{1,\dots ,N\}$ we choose $\varphi _{\varepsilon ,\delta }=\xi _\varepsilon \Psi _\delta e_i$ as testing function in the weak formulation (1.5), where $e_i$ denotes the *i*-th canonical basis vector in $\mathbb {R}^N$. In the limit $\varepsilon ,\delta \downarrow 0$ we obtainWe multiply the preceding inequality by $e_i$ and sum over $i=1,\dots ,N$. This yieldsHere, we use the growth condition (1.4)$_2$ and immediately get for any $t_1,t_2\in \Lambda _\varrho ^{(\lambda ,\theta )}(t_o)$ and any $r \in [\frac{\varrho }{2}, \varrho ]$ that there holdsSince

$$\begin{aligned} \int _{t_1}^{t_2}\int _{B_\varrho (x_o)} \big [L|Du|^{p-1} + |F|\big ] \,\mathrm {d}x\mathrm {d}t&= \int _0^\varrho \int _{t_1}^{t_2}\int _{\partial B_{r}(x_o)} \big [L|Du|^{p-1} + |F|\big ] \,\text {d}\mathcal {H}^{n-1}\mathrm {d}t\mathrm {d}r\\&\ge \int _{\varrho /2}^\varrho \int _{t_1}^{t_2}\int _{\partial B_{r}(x_o)} \big [L|Du|^{p-1} + |F|\big ] \,\text {d}\mathcal {H}^{n-1}\mathrm {d}t\mathrm {d}r, \end{aligned}$$

there exists a radius ${\hat{\varrho }} \in [\frac{\varrho }{2},\varrho )$ with

$$\begin{aligned} \int _{t_1}^{t_2}\int _{\partial B_{{\hat{\varrho }}}(x_o)} \big [L|Du| ^{p-1}+ |F|\big ] \,\text {d}\mathcal {H}^{n-1}\mathrm {d}t\le \tfrac{2}{\varrho } \int _{t_1}^{t_2}\int _{B_\varrho (x_o)} \big [L|Du| ^{p-1}+ |F|\big ] \,\mathrm {d}x\mathrm {d}t. \end{aligned}$$

Therefore, we choose in the above inequality $r={\hat{\varrho }}$ and then take means on both sides of the resulting inequality. This impliesfor any $t_1,t_2\in \Lambda _\varrho ^{(\theta )}(t_o)$ and with a constant $c=c(L)$. $\square$

## Parabolic Sobolev–Poincaré type inequalities

In this section we consider cylinders on which certain intrinsic or sub-intrinsic couplings with respect to *u* and its spatial gradient *Du* are satisfied. In order to cover all the needed cases of intrinsic and sub-intrinsic couplings, we now formulate the conditions that appear later in the course of the work. Later on, we will explicitly state which of the conditions must be satisfied. On a scaled cylinder $Q_{2\varrho }^{(\lambda ,\theta )}(z_o)\subseteq \Omega _T$ with radius $0<\varrho \le 1$ and scaling parameters $\lambda ,\theta >0$ we introduce couplings of the type4.1and4.2with $p^\sharp {:}{=}\max \{p,q+1\}$ and some constants $C_\lambda ,\, C_\theta \ge 1$. These inequalities are to be interpreted as follows. Assume that (4.1)$_1$ resp. (4.2)$_1$ is valid on $Q_{2\varrho }^{(\lambda ,\theta )}(z_o)\subseteq \Omega _T$ for a constant $C_\lambda$ or $C_\theta$. Then we call the scaled cylinder $\lambda$-sub-intrinsic (with constant $C_\lambda$) or $\theta$-sub-intrinsic (with constant $C_\theta$) . Similarly, if (4.1)$_2$ resp. (4.2)$_2$ holds, then we call $Q_{\varrho }^{(\lambda ,\theta )}(z_o)$ $\lambda$-super-intrinsic (with constant $C_\lambda$) or $\theta$-sub-intrinsic (with constant $C_\theta$). Cylinders which are both $\lambda$-sub-intrinsic and $\lambda$-super-intrinsic are called $\lambda$-intrinsic. We use the same notation for $\theta$-sub-intrinsic and $\theta$-super-intrinsic cylinders. When we speak of a (sub-, super-) intrinsic coupling, we implicitly assume that a constant $C_\lambda$ or $C_\theta$ exists, so that the respective coupling holds. Note that (4.2)$_1$ immediately implies4.3i.e. the subintrinsic coupling holds on $Q_{\varrho }^{(\lambda ,\theta )}(z_o)$ with constant $2^{n+p^\sharp +q+1}C_\theta$. A similar observation can be made for the case of (4.1)$_1$, i.e. if (4.1)$_1$ holds, then4.4The following corollary is an immediate consequence of Lemma  3.2.

### Corollary 4.1

Let $p>1$ and $0<q\le 1$, and let *u* be a weak solution to (1.3) in $\Omega _T$ in the sense of Definition  1.1 and consider a cylinder $Q_{2\varrho }^{(\lambda ,\theta )}(z_o)\subseteq \Omega _T$ with $0<\varrho \le 1$ and $\lambda ,\theta >0$, satisfying the $\theta$-sub-intrinsic condition (4.2)$_1$. Moreover, for $r\in (0,\varrho )$ let $a_r:[0,T]\rightarrow \mathbb {R}^N$ be given by , where  denotes the slice-wise mean of  on $B_\varrho (x_o)$ as defined in (2.3). Then, there exists ${\hat{\varrho } \in } [\frac{\varrho }{2},\varrho ]$ such that for all $t_1,t_2\in \Lambda _\varrho ^{(\lambda ,\theta )}(t_o)$ there holdsprovided $\max \{\frac{1}{p},\frac{p-1}{p}\}\le \vartheta \le 1$, where $c=c(n,p,q,L,C_\theta )$.

### Proof

For simplification we shall omit the reference point $z_o$ throughout the proof in our notation. In turn, we apply Lemma  2.2 with $\alpha =\frac{1}{q}$ and Lemma  3.2, to infer that there exists ${\hat{\varrho \in }} [\frac{\varrho }{2},\varrho ]$ such that for a.e. $t,\tau \in \Lambda _\varrho ^{(\lambda , \theta )}$ there holdsfor a constant *c* depending only on *n*, *p*, *q* and *L*. We take both sides to the power $\vartheta p$, integrate with respect to *t* and $\tau$ over $\Lambda ^{\lambda ,\theta }_\varrho$, and then apply Hölder’s inequality, where we use $0<q<1<p$. Then, using the very definition of $\tau _\varrho ^{(\lambda ,\theta )}$ and the sub-intrinsic coupling (4.2)$_1$ (in a form similar to (4.3) with ${\hat{\varrho }}$ instead of $\varrho$), we find thatwith a constant $c=c(n,p,q,L)$. This proves the claim. $\square$

The next lemma is a Poincaré type inequality for weak solutions. Corollary 4.1 will serve to overcome the lack of differentiability with respect to the time variable.

### Lemma 4.2

Let $p>1$ and $0<q\le 1$, and let *u* be a weak solution to (1.3) in $\Omega _T$ in the sense of Definition 1.1. Then, on any cylinder $Q_{2\varrho }^{(\lambda ,\theta )}(z_o)\subseteq \Omega _T$ with $0<\varrho \le 1$ and $\lambda ,\theta >0$ satisfying the $\theta$-sub-intrinsic coupling (4.2)$_1$, the following Poincaré type inequalityholds true for any $\max \{\frac{1}{p},\frac{p-1}{p}\}\le \vartheta \le 1$ and a universal constant $c=c(n,p,q,L,C_\theta )$.

### Proof

In the following we shall omit the reference point $z_o$ in our notation. We also use the abbreviationBy ${\hat{\varrho } \in } [\frac{\varrho }{2},\varrho ]$ we denote the radius from Corollary 4.1. We now define the slice-wise means  of  as in (2.3) and $a_r:[0,T]\rightarrow \mathbb {R}^N$ by  for any $t\in [0,T]$. By adding and subtracting $a_{{\hat{\varrho }}}(t)$, we obtain4.5with the obvious meaning of the abbreviations $\hbox {I}$ – $\hbox {III}$. In the following, we treat the terms of the right side one after the other. We start with the term I. We apply Lemma 2.4 with the choices $\alpha =\frac{1}{q}$, $A=B_{{\hat{\varrho }}}$, $B=B_\varrho$,  and with  in place of *u*. Since $|B|/|A|\le 2^n$, we obtainwith $c=c(n,p,q)$. In the last step, we used Poincaré’s inequality slice-wise. A similar argument applies to the third term $\hbox {III}$, since $\hbox {III}\le \hbox {I}$. The bound for II directly follows from Corollary 4.1. Indeed, we havefor some constant *c* depending only on *n*, *p*, *q* and *L*. The application of Corollary 4.1 is permitted, since we have assumed the $\theta$-sub-intrinsic coupling (4.2)$_1$. At this point, we use the estimates for I, II, and III in (4.5) and arrive atThis yields the claim. $\square$

The following lemma can be interpreted as a Sobolev–Poincaré type inequality for weak solutions.

### Lemma 4.3

Let $p>1$ and $0<q\le 1$, and let *u* be a weak solution to (1.3) in $\Omega _T$ in the sense of Definition  1.1. Then, on any cylinder $Q_{2\varrho }^{(\lambda ,\theta )}(z_o)\subseteq \Omega _T$ satisfying the $\lambda$-intrinsic coupling (4.1) and the $\theta$-sub-intrinsic coupling (4.2)$_1$ for some $0<\varrho \le 1$ and some $\lambda , \theta >0$, the following inequalityholds true for any given $\epsilon \in (0,1]$ and universal constants $c=c(n,p,q,L,C_\theta ,C_\lambda )$ and $\beta =\beta (n,p,q)$. Here, the parameter $\vartheta$ is defined by $\vartheta {:}{=}\max \big \{\frac{n}{n+q+1},\frac{1}{p}, \frac{p-1}{p}\big \}.$

### Proof

In the following we shall omit again the reference point $z_o$ in our notation and abbreviate $a{:}{=}(u)_{\varrho }^{(\lambda ,\theta )}$. Moreover, we use the short-hand notations4.6With Lemma 2.4 and the $\theta$-sub-intrinsic coupling (4.2)$_1$ in the form of (4.3) we conclude the bound4.7It suffices to consider the case $\mathbf{E}>\mathbf{F}$, since otherwise, the asserted estimate is clearly satisfied. Under this assumption, estimate (4.7) implies

4.8 $$\begin{aligned} \mathbf{F}\le c\,\theta ^p \end{aligned}$$

with a constant $c=c(n,p,q,C_\theta )$. Moreover, Hölder’s inequality and the $\lambda$-sub-intrinsic coupling in the form (4.4) imply4.9where $c=c(n,C_\lambda )$. Next, we apply Gagliardo-Nirenberg’s inequality as stated in Lemma 2.6, with $(p,q,r,\theta )$ replaced by $(p,\vartheta p,q+1,\vartheta )$, which is possible since $\vartheta \ge \max \{\frac{n}{n+q+1},\frac{1}{p}\}$. Afterwards, we use Lemma 4.2. In this way we find4.10with a constant $c=c(n,p,q,L,C_\theta )$. Next, we use Lemma 2.2 with $\alpha =\frac{q+1}{2}$ for the estimateWe divide this inequality by $\varrho ^{q+1}$ and take the mean value integral over $B_\varrho$. Then we use Hölder’s inequality and estimate $|a|=|(u)_\varrho ^{(\lambda ,\theta )}|$ by means of the $\theta$-sub-intrinsic coupling in the form (4.3). This leads to the bound4.11with a constant $c=c(q,C_\theta )$. In the second to last line, we used the definition of $\mathbf{S}$ and the definition of $\tau _\varrho ^{(\lambda ,\theta )}$ according to (2.1), whereas in the last line we used inequality (4.8). Using this inequality to bound the right-hand side of (4.10), we arrive at the estimate

4.12 $$\begin{aligned} \mathbf{E}&\le c\big [\lambda ^{2-p}\mathbf{F}^{\frac{q-1}{p}}\,\mathbf{S}\big ]^{\frac{(1-\vartheta )p}{q+1}} \Big [\mathbf{F}^\vartheta + \lambda ^{\vartheta p(2-p)}\mathbf{F}^{\vartheta (p-1)}\Big ]\nonumber \\&\quad + c\big [\lambda ^{2-p}\,\mathbf{S}\big ]^{\frac{(1-\vartheta )p}{2}} \Big [\mathbf{F}^\vartheta + \lambda ^{\vartheta p(2-p)}\mathbf{F}^{\vartheta (p-1)}\Big ] \end{aligned}$$

with a constant $c=c(n,p,q,L,C_\theta )$. In the case $p\ge 2$, we can use (4.9) to bound the negative powers of $\lambda$ from above by powers of $\mathbf{F}$, leading us to

$$\begin{aligned} \mathbf{E}&\le c\,\mathbf{S}^{\frac{(1-\vartheta )p}{q+1}} \mathbf{F}^{\frac{1+q-(1-\vartheta )p}{q+1}} + c\,\mathbf{S}^{\frac{(1-\vartheta )p}{2}} \mathbf{F}^{\frac{2-(1-\vartheta )p}{2}}\nonumber \\&\le \varepsilon \,\mathbf{S}+c\varepsilon ^{-\frac{(1-\vartheta )p}{1+q-(1-\vartheta )p}}\,\mathbf{F}, \end{aligned}$$

for every $\varepsilon \in (0,1]$ with a constant $c=c(n,p,q,L,C_\theta ,C_\lambda )$, where the last estimate follows by two applications of Young’s inequality. Finally, we note that the exponent of $\varepsilon$ is bounded from below by $-\frac{1}{q}$, since $\vartheta \ge \frac{p-1}{p}$. This yields the asserted inequality for exponents $p\ge 2$.

In the remaining case $p<2$, we note that (4.9) implies

$$\begin{aligned} \mathbf{F}^\vartheta \le c\lambda ^{\vartheta p(2-p)}\mathbf{F}^{\vartheta (p-1)} \end{aligned}$$

with a constant $c=c(n,p,q,C_\lambda )$. In this way, we deduce

4.13 $$\begin{aligned} \mathbf{E}&\le c\big [\lambda ^{2-p}\,\mathbf{S}\big ]^{\frac{(1-\vartheta )p}{q+1}} \lambda ^{\vartheta p(2-p)}\mathbf{F}^{\vartheta (p-1)-\frac{(1-q)(1-\vartheta )}{q+1}}\nonumber \\&\qquad + c\big [\lambda ^{2-p}\,\mathbf{S}\big ]^{\frac{(1-\vartheta )p}{2}} \lambda ^{\vartheta p(2-p)}\mathbf{F}^{\vartheta (p-1)}\nonumber \\&= c\,\mathbf{S}^{\frac{(1-\vartheta )p}{q+1}} \lambda ^{\frac{p(2-p)(1+\vartheta q)}{q+1}} \mathbf{F}^{\frac{\vartheta (pq+p-2q)-(1-q)}{q+1}}\nonumber \\&\qquad + c\,\mathbf{S}^{\frac{(1-\vartheta )p}{2}} \lambda ^{\frac{p(2-p)(1+\vartheta )}{2}}\mathbf{F}^{\vartheta (p-1)}\nonumber \\&\le \varepsilon \big (\mathbf{S}+\lambda ^p\big ) + c\varepsilon ^{-\frac{2-\vartheta (pq+p-2q)}{\vartheta (pq+p-2q)-(1-q)}}\mathbf{F}\end{aligned}$$

for every $\varepsilon \in (0,1]$, with a constant $c=c(n,p,q,L,C_\theta ,C_\lambda )$. In the last line, we applied Young’s inequality for three factors, once with exponents $\frac{q+1}{(1-\vartheta )p}$, $\frac{q+1}{(2-p)(1+\vartheta q)}$, and $\frac{q+1}{\vartheta (pq+p-2q)-(1-q)}$, and once with $\frac{2}{(1-\vartheta )p}$, $\frac{2}{(2-p)(1+\vartheta )}$, and $\frac{1}{\vartheta (p-1)}$. Using $\vartheta \ge \frac{1}{p}$ and $p<2$, we can estimate the exponent of $\varepsilon$ from (4.13) from below by $-\frac{1}{q(p-1)}$. Since $\lambda ^p$ can be estimated from above with the help of the $\lambda$-super-intrinsic coupling condition (4.1)$_2$, estimate (4.13) implies the claim in the remaining case $p<2$. $\square$

Moreover, we need a version of the Sobolev–Poincaré inequality to control the term in the energy estimate that stems from the time term of the parabolic equation.

### Lemma 4.4

Let $p>\frac{2n}{n+2}$ and $0<q\le 1$, and let *u* be a weak solution to (1.3) in $\Omega _T$ in the sense of Definition 1.1. Then, on any cylinder $Q_{2\varrho }^{(\lambda ,\theta )}(z_o)\subseteq \Omega _T$ satisfying the $\lambda$-intrinsic coupling (4.1) and the $\theta$-sub-intrinsic coupling (4.2)$_1$ for some $0<\varrho \le 1$ and some $\lambda ,\theta >0$, the following inequalityholds true for any given $\varepsilon \in (0,1]$ and universal constants $c=c(n,p,q,L,C_\theta ,C_\lambda )$ and $\beta =\beta (n,p,q)>0$. Here, the parameter $\vartheta$ is defined by $\vartheta {:}{=}\frac{n}{n+2}.$

### Proof

We omit again the reference point $z_o$ and write $a{:}{=}(u)_\varrho ^{(\lambda ,\theta )}$ and continue to use the abbreviations introduced in (4.6). We begin with the case $\frac{2n}{n+2}<p\le 2$. We split off a certain power of the spatial integrand and estimate it by the supremum over the time-slices. Then we use the estimate $\frac{|a|}{\varrho }\le c\theta$, which follows by the sub-intrinsic coupling in the form (4.3) and apply Lemma 2.2. Finally, we apply the Sobolev inequality. This procedure leads to the estimate4.14with a constant $c=c(n,p,q,L,C_\theta )$. We estimate the last integral by means of Lemma 4.2 with $\vartheta =\frac{2n}{p(n+2)}$ and arrive at

$$\begin{aligned} \mathbf{L} \le c\lambda ^{\frac{n(p-2)}{n+2}}\mathbf{S}^{\frac{2}{n+2}} \Big [\mathbf{F}^\vartheta + \lambda ^{\vartheta p(2-p)}\mathbf{F}^{\vartheta (p-1)}\Big ]. \end{aligned}$$

In the last term we use the estimate $\mathbf{F}\le c\lambda ^p$, which follows from the $\lambda$-sub-intrinsic coupling in the form (4.4). Then, we apply Young’s inequality with exponents $\frac{n+2}{2}$, $\frac{p(n+2)}{n(2-p)}$, and $\frac{p(n+2)}{2n(p-1)}$ and obtain the bound

$$\begin{aligned} \mathbf{L}&\le c\,\mathbf{S}^{\frac{2}{n+2}} \lambda ^{\frac{n(2-p)}{n+2}} \mathbf{F}^{\vartheta (p-1)}\\&\le \varepsilon (\mathbf{S}+\lambda ^p) +c\varepsilon ^{-\frac{2p+n(2-p)}{2n(p-1)}}\mathbf{F}, \end{aligned}$$

for every $\varepsilon \in (0,1]$, with $c=c(n,p,q,\nu ,L,C_\lambda ,C_\theta )$. Since $\lambda$ can be bounded from above by the $\lambda$-super-intrinsic coupling condition (4.1)$_2$, this yields the claim in the case $\frac{2n}{n+2}<p\le 2$.

For exponents $p>2$, we use Lemma 2.2 and then use Young’s inequality to obtainNow the claim follows by an application of Lemma 4.3. $\square$

Finally, we prove a third version of a Sobolev–Poincaré inequality that will be necessary in the case $p<q+1$. We note that in the case $p\ge q+1$, it could be replaced by Lemma 4.3. However, we state the lemma for all cases in order to reduce the case distinctions in the further proof to a minimum.

### Lemma 4.5

Let $p>\frac{n(q+1)}{n+q+1}$ and $0<q\le 1$, and *u* be a weak solution to (1.3) in $\Omega _T$ in the sense of Definition 1.1. Then, on any cylinder $Q_{2\varrho }^{(\theta )}(z_o)\subseteq \Omega _T$ satisfying the $\lambda$-intrinsic coupling (4.1) and the $\theta$-sub-intrinsic coupling (4.2)$_1$ for some $0<\varrho \le 1$ and some $\lambda ,\theta >0$, the following inequalityholds true for any given $\varepsilon \in (0,1]$ and universal constants $c=c(n,p,q,L,C_\theta ,C_\lambda )$, $\beta =\beta (n,p)>0$, and the parameter $\vartheta {:}{=}\max \big \{\frac{n(q+1)}{p(n+q+1)},\frac{n}{n+q+1},\frac{1}{p}, \frac{p-1}{p}\big \}.$

### Proof

We again omit the reference point $z_o$ and abbreviate $a{:}{=}(u)_{\varrho }^{(\lambda ,\theta )}$ andMoreover, we continue to use the abbreviations $\mathbf{F}$ and $\mathbf{S}$ introduced in (4.6). First, we consider the case $p<q+1$, in which $p^\sharp =q+1$. We start with the observation that the $\theta$-sub-intrinsic coupling in the form of (4.3) implieswith a constant $c=c(n,p,q,C_\theta )$. Moreover, we can assume that

$$\begin{aligned} \lambda ^{p-2}\widetilde{\mathbf {E}}^{\frac{2}{q+1}}>\mathbf{F}, \end{aligned}$$

since otherwise, the assertion is obviously true. The combination of the two preceding inequalities yields

4.15 $$\begin{aligned} \mathbf{F}\le c\lambda ^{p-2}\theta ^ 2, \end{aligned}$$

where $c=c(n,p,q,C_\theta )$. For the proof of the Sobolev type inequality, we estimate a part of the spatial integral in $\widetilde{\mathbf {E}}$ by its supremum over time and apply Lemma 2.2 to the other part. In a second step, we apply Sobolev’s inequality, with the resultWe estimate the last term by Lemma 4.2 with $\vartheta =\frac{n(q+1)}{p(n+q+1)}$ and then the $\lambda$-sub-intrinsic coupling in the form (4.4), which implies $\mathbf{F}\le c\lambda ^p$. This leads to the bound

$$\begin{aligned} \widetilde{\mathbf {E}}&\le c\big [\lambda ^{2-p}\theta ^{q-1}\big ]^{\frac{q+1}{n+q+1}}\mathbf{S}^{\frac{q+1}{n+q+1}} \Big [\mathbf{F}^\vartheta + \lambda ^{\vartheta p(2-p)}\mathbf{F}^{\vartheta (p-1)}\Big ]\\&\le c\big [\lambda ^{2-p}\theta ^{q-1}\big ]^{\frac{q+1}{n+q+1}}\mathbf{S}^{\frac{q+1}{n+q+1}} \lambda ^{\vartheta p(2-p)}\mathbf{F}^{\vartheta (p-1)} \end{aligned}$$

with $c=c(n,p,q,\nu ,L,C_\lambda ,C_\theta )$. We take the preceding estimate to the power $\frac{2}{q+1}$ and multiply both sides with $\lambda ^{p-2}$. In this way, we infer

$$\begin{aligned} \lambda ^{p-2}\widetilde{\mathbf {E}}^{\frac{2}{q+1}}&\le c\lambda ^{\frac{n(2-p)}{n+q+1}}\big [\lambda ^{p-2}\theta ^2\big ]^{\frac{q-1}{n+q+1}} \mathbf{S}^{\frac{2}{n+q+1}} \mathbf{F}^{\frac{2n(p-1)}{p(n+q+1)}}. \end{aligned}$$

Next, we use the lower bound (4.15) for $\lambda ^{p-2}\theta ^2$, together with the fact $q\le 1$. Then we apply Young’s inequality with exponents $\frac{p(n+q+1)}{n(2-p)}$, $\frac{n+q+1}{2}$ and $\frac{p(n+q+1)}{2n(p-1)+p(q-1)}$ and obtain

$$\begin{aligned} \lambda ^{p-2}\widetilde{\mathbf {E}}^{\frac{2}{q+1}}&\le c\lambda ^{\frac{n(2-p)}{n+q+1}}\mathbf{S}^{\frac{2}{n+q+1}} \mathbf{F}^{\frac{2n(p-1)+p(q-1)}{p(n+q+1)}}\\&\le \varepsilon (\mathbf{S}+\lambda ^p)+c\varepsilon ^{-\beta }\mathbf{F}, \end{aligned}$$

for every $\varepsilon \in (0,1]$, with constants $c=c(n,p,q,\nu ,L,C_\lambda ,C_\theta )$ and $\beta =\beta (n,p,q)>0$. This yields the claim in the case $p<q+1$, since $\lambda ^p$ can be bounded from above by (4.1). In the remaining case $p\ge q+1$, in which $p^\sharp =p$, the asserted estimate is a consequence of Lemma 4.3. More precisely, Lemma 2.2 implies4.16In the case $q+1\le p<2$, we use Lemma 4.2 and the $\lambda$-sub-intrinsic coupling in the form (4.4) to estimatewith $c=c(n,p,q,L,C_\lambda )$. Combining the preceding estimates, we concludefrom which the assertion follows in the case $q+1\le p<2$ by estimating the last integral with the help of Lemma 4.3. In the case $p\ge 2\ge q+1$, we apply Young’s inequality to the right-hand side of (4.16) and deducefor every $\delta \in (0,1]$. Estimating the last term by Lemma 4.3, we deducefor any $\delta ,\kappa \in (0,1]$, with $\vartheta {:}{=}\max \big \{\frac{n}{n+q+1},\frac{1}{p}, \frac{p-1}{p}\big \}$, $c=c(n,p,q,\nu ,L,C_\lambda ,C_\theta )$ and $\alpha =\alpha (n,p,q)>0$. This implies the claim in the remaining case by choosing $\delta =\frac{\varepsilon }{2}$ and $\kappa =\frac{1}{2}\varepsilon \delta ^{\frac{p-2}{2}}$. $\square$

### Lemma 4.6

Let $p>\frac{n(q+1)}{n+q+1}$ and $0<q\le 1$, and *u* be a weak solution to (1.3) in $\Omega _T$ in the sense of Definition 1.1. Then, on any cylinder $Q_{2\varrho }^{(\theta )}(z_o)\subseteq \Omega _T$ that satisfies the $\lambda$-intrinsic coupling (4.1) and the $\theta$-intrinsic coupling (4.2) with constant $C_\theta =1$, for some $0<\varrho \le 1$ and some $\lambda , \theta >0$, we havewhere $c=c(n,p,q,\nu ,L,C_\lambda )$.

### Proof

Again, we omit the center $z_o$ in the notation and write $a_\sigma =(u)_{\sigma }^{(\lambda ,\theta )}$ for $\sigma \in \{\frac{\varrho }{2},\varrho \}$. We use the $\theta$-super-intrinsic coupling (4.2)$_2$ with constant $C_\theta =1$, Minkowski’s inequality and finally, Lemma 2.4. This yields the bound4.17where $c=c(p,q)$. For the estimate of the second last integral, we distinguish between the cases $p\ge q+1$ and $p<q+1$. In the first case, in which $p^\sharp =p$, we use in turn Lemma 2.3, Lemma 4.2 with $\vartheta =1$, and then the $\lambda$-sub-intrinsic coupling (4.1)$_1$ to deduce4.18with $c=c(n,p,q,L,C_\lambda )$. In the case $p<q+1$, we use Lemma 4.5 instead. In view of the $\lambda$-sub-intrinsic coupling (4.1)$_1$ we inferfor every $\varepsilon \in (0,1]$, with $c=c(n,p,q,L,C_\lambda )$ and $\beta =\beta (n,p,q)>0$. We estimate the supremum on the right-hand side by the energy estimate from Lemma 3.1 and then by Lemma 2.4 with $a=0$ and obtainwith $c=c(p,q,\nu ,L,C_\lambda )$, where we applied the coupling conditions (4.1)$_1$ and (4.2)$_2$ in the last step. Joining the two preceding estimates and applying Young’s inequality, we infer4.19which holds in the case $p<q+1$. Using (4.18), respectively (4.19) in (4.17), we deduce that in both cases the estimateholds for every $\varepsilon \in (0,1]$, with $c=c(n,p,q,\nu ,L,C_\lambda )$ and $\beta =\beta (n,p,q)$. At this stage, we choose the parameter $\varepsilon \in (0,1]$ in dependence on $n,p,q,\nu ,L,$ and $C_\lambda$ so small that

$$\begin{aligned} 1-c\varepsilon ^{\frac{q+1}{2}}\ge 2^{-\frac{q}{2}}. \end{aligned}$$

This allows us to absorb the first term from the right-hand side into the left-hand side. After dividing by $1-c\varepsilon ^{\frac{q+1}{2}}$, we arrive at the boundwith $c=c(n,p,q,\nu ,L,C_\lambda )$. This completes the proof of the lemma. $\square$

## Reverse Hölder inequality

### Proposition 5.1

Let $p>\frac{2n}{n+2}$ and $0<q\le 1$, and *u* be a weak solution to (1.3) in $\Omega _T$ in the sense of Definition 1.1. Then, on any cylinder $Q_{2\varrho }^{(\lambda , \theta )}(z_o)\Subset \Omega _T$ that satisfies the $\lambda$-intrinsic coupling (4.1) and the $\theta$-intrinsic coupling (4.2) for some $0<\varrho \le 1$, $\lambda \ge 1$ and $\theta >0$, the following reverse Hölder type inequality holds true5.1for $\vartheta {:}{=}\max \big \{\frac{n(q+1)}{p(n+q+1)},\frac{n}{n+q+1},\frac{1}{p}, \frac{p-1}{p}\big \}$ and some universal constant *c* that depends on $n,p,q,\nu , L,C_\lambda ,$ and $C_\theta$.

### Proof

Once again, we omit the reference to the center $z_o$ in the notation. We consider radii *r*, *s* with $\varrho \le r<s\le 2\varrho$ and write $a_\sigma {:}{=}(u)_\sigma ^{(\lambda ,\theta )}$ with $\sigma \in \{r,s\}$. From the energy estimate in Lemma  3.1 we obtain5.2with the obvious abbreviation of I–III. The dependencies of the constant are given by $c=c(p,q,\nu ,L)$. With the $\theta$-super-intrinsic coupling (4.2)$_2$ we first estimate5.3We introduce the abbreviation

5.4 $$\begin{aligned} {\mathcal {R}}_{r,s} {:}{=} \frac{s}{s-r}. \end{aligned}$$

For the estimate of the first term in (5.2), we use in turn (5.3) and the assumption $0<q\le 1$, then Hölder’s inequality and finally, Lemma 2.4 to replace $a_r$ by $a_s$. This procedure results in the estimatewith $c=c(n,p,q,\nu ,L,C_\theta )$. In the term $\mathrm {II}$, we also apply Lemma 2.4 and obtain5.5Collecting the estimates and using Lemmas 4.3, 4.4, and 4.5 to estimate the individual terms, we arrive atfor any $\varepsilon \in (0,1]$, with $c=c(n,p,q,\nu ,L,C_\lambda ,C_\theta )$. Here, we choose $\varepsilon =\frac{1}{2c{\mathcal {R}}_{r,s}^{p^\sharp }}$ and obtainTo re-absorb the term $\frac{1}{2}(\dots )$ from the right-hand side into the left-hand side, we apply the Iteration Lemma 2.1. This leads to the claimed reverse Hölder type inequality (5.1) and finishes the proof of Proposition  5.1. $\square$

### Proposition 5.2

Let $p>\frac{2n}{n+2}$ and $0<q\le 1$, and *u* be a weak solution to (1.3) in $\Omega _T$ in the sense of Definition 1.1. Then, on any cylinder $Q_{2\varrho }^{(\lambda ,\theta )}(z_o)\Subset \Omega _T$ satisfying the $\lambda$-intrinsic coupling (4.1) and a coupling condition of the form5.6for some $0<\varrho \le 1$, $\lambda \ge 1$ and $\theta >0$ and some constants $C_\theta , K_\theta \ge 1$, the following reverse Hölder type inequality holds true5.7with $\vartheta {:}{=}\max \big \{\frac{n(q+1)}{p(n+q+1)},\frac{n}{n+q+1},\frac{1}{p}, \frac{p-1}{p}\big \}$, and where the constant *c* depends on *n*, *p*, *q*, $\nu ,L,C_\lambda ,C_\theta ,$ and $K_\theta$.

### Proof

The largest part of the proof is identical to that of Proposition 5.1. We again start with the energy estimate (5.2) and estimate the term $\mathrm {II}$ therein by (5.5). The only difference is the estimation of $\mathrm {I}$, which is now based on (5.6). More precisely, we use (5.6) in combination with (4.4) to deduce

$$\begin{aligned} \theta ^p\le 2^{n+q+1}K_\theta C_\lambda \lambda ^p. \end{aligned}$$

For the estimate of $\mathrm {I}$, we use in turn the preceding estimate, then Hölder’s inequality, Lemma 2.4 and finally, Young’s inequality with exponents $\frac{2}{1-q}$ and $\frac{2}{q+1}$ to inferwith a constant $c=c(n,p,q,\nu ,L,C_\lambda ,K_\theta )$. Using this estimate in (5.2) and recalling (5.5), we arrive at5.8The first term on the right-hand side can be estimated with the help of the $\lambda$-super-intrinsic coupling (4.1)$_2$ in the formand the integral of $|Du|^p$ can be absorbed into the left-hand side. Next, we estimate the first integral on the right-hand side of (5.8) by Lemma 4.5 and the second one by Lemma 4.3 and obtain the boundfor any $\varepsilon \in (0,1]$, with $c=c(n,p,q,\nu ,L,C_\lambda ,C_\theta ,K_\theta )$. In the preceding estimate, we choose the parameter $\varepsilon$ in the form $\varepsilon =\frac{1}{2c{\mathcal {R}}_{r,s}^{2\vee p}}$ and arrive atwhere $c=c(n,p,q,\nu ,L,C_\lambda ,C_\theta ,K_\theta )$. Now, we apply the Iteration Lemma 2.1 to re-absorb the first two terms on the right-hand side into the left. This yields the claimed reverse Hölder inequality (5.7) and finishes the proof of the Proposition. $\square$

## Proof of the higher integrability

We consider a fixed cylinder $Q_{4R} \equiv Q_{4R}^{(1,1)}$ with $R\in (0,1]$ such that $Q_{8R}\subset \Omega _T$ and some6.1with the scaling deficit *d* introduced in (1.8), i.e.

$$\begin{aligned} d= \left\{ \begin{array}{cl} \frac{p}{q+1},&{}\hbox {for }p\ge q+1,\\ \frac{p(q+1)}{p(q+1)+n(p-q-1)},&{}\hbox {for }\frac{2n}{n+2}<p<q+1. \end{array} \right. \end{aligned}$$

Moreover, we fix $\lambda \ge \lambda _o$ and define $R_o{:}{=}\min \{1,\lambda ^{\frac{p-q-1}{q+1}}\}R$. Then, for any $z_o\in Q_{2R}$, any $\theta \ge \lambda$ and every $\varrho \le R_o$, we have $Q_{2\varrho }^{(\lambda ,\theta )}(z_o)\subset Q_{4R}$.

### Construction of a non-uniform system of cylinders

The following construction of a non-uniform system of cylinders is similar to the one in [11]. Let $z_o\in Q_{2R}$. For a radius $\varrho \in (0,R_o]$ we now define

$$\begin{aligned} {\widetilde{\theta }}_{z_o;\varrho }^{(\lambda )} {:}{=} \inf \bigg \{\theta \in [\lambda ,\infty ): \frac{1}{|Q_\varrho |} \iint _{Q_{\varrho }^{(\lambda ,\theta )}(z_o)} \frac{|u|^{p^\sharp }}{\varrho ^{p^\sharp }} \,\mathrm {d}x\mathrm {d}t\le \lambda ^{2-p}\theta ^{p^\sharp +q-1} \bigg \}, \end{aligned}$$

where we again use the notation $p^\sharp =\max \{p,q+1\}$. If the meaning is clear from the context we write ${\widetilde{\theta }}_\varrho$ instead of ${\widetilde{\theta }}_{z_o;\varrho }^{(\lambda )}$. Of course, this assumes that $z_o$ and $\lambda$ are fixed in the respective context. We note that the set of those $\theta \ge \lambda$ for which the condition above is satisfied is obviously not empty. In fact, in the limit $\theta \uparrow \infty$, by definition of $Q_{\varrho }^{(\lambda ,\theta )}(z_o)$ the integral converges to zero in the case $0<q<1$ and stays bounded for $q=1$, while the right-hand side blows up with speed $\theta ^{p^\sharp +q-1}$.

Observe that the condition in the infimum can be re-written in the formTherefore, we either haveor otherwise6.2Note that ${\widetilde{\theta }}_{R_o}\ge \lambda \ge \lambda _o\ge 1$. If $\lambda <{\widetilde{\theta }}_{R_o}$, then by (6.2) we haveTherefore, in any case we end up with the bound

6.3 $$\begin{aligned} {\widetilde{\theta }}_{R_o} \le 4^{\frac{n+p^\sharp +q+1}{p^\sharp +q-1}}\lambda . \end{aligned}$$

Next, we establish that the mapping $(0,R_o]\ni \varrho \mapsto {\widetilde{\theta }}_\varrho$ is continuous. To this end, consider $\varrho \in (0,R_o]$ and $\varepsilon >0$, and define $\theta _+{:}{=}{\widetilde{\theta }}_\varrho +\varepsilon$. Then, there exists $\delta =\delta (\varepsilon ,\varrho )>0$ such that

$$\begin{aligned} \frac{1}{|Q_r|}\iint _{Q_{r}^{(\lambda , \theta _+)}(z_o)} \frac{|u|^{p^\sharp }}{r^{p^\sharp }} \,\mathrm {d}x\mathrm {d}t< \lambda ^{2-p}\theta _{+}^{p^\sharp +q-1} \end{aligned}$$

for any $r\in (0,R_o]$ with $|r-\varrho |<\delta$. In fact, the preceding strict inequality holds by the very definition of ${\widetilde{\theta }}_\varrho$ with $r=\varrho$, since the integral on the left-hand side decreases with the replacement of ${\widetilde{\theta }}_\varrho$ by $\theta _+$ (note that $\Lambda _{\varrho }^{(\lambda , \theta _+)}(t_o) \subset \Lambda _{\varrho }^{(\lambda , {\widetilde{\theta }}_\varrho )}(t_o)$, so that the integral decreases), while the right-hand side strictly increases. The claim now follows, since both the integral on the right and the left-hand side are continuous functions with respect to the radius *r*. In other words, we have shown that ${\widetilde{\theta }}_r\le \theta _+={\widetilde{\theta }}_\varrho +\varepsilon$ for radii *r* as above. It remains to prove ${\widetilde{\theta }}_r\ge \theta _-{:}{=}{\widetilde{\theta }}_\varrho -\varepsilon$ for *r* close to $\varrho$. If $\theta _-\le \lambda$ holds, then ${\widetilde{\theta }}_r\ge \lambda \ge \theta _-$ for any *r*. In the other case, after diminishing $\delta =\delta (\varepsilon ,\varrho )>0$ if necessary, we get

$$\begin{aligned} \frac{1}{|Q_r|} \iint _{Q_{r}^{(\lambda , \theta _-)}(z_o)} \frac{|u|^{p^\sharp }}{r^{p^\sharp }} \,\mathrm {d}x\mathrm {d}t> \lambda ^{2-p} \theta _-^{p^\sharp +q-1} \end{aligned}$$

for all $r\in (0,R_o]$ with $|r-\varrho |<\delta$. For $r=\varrho$, this is a direct consequence of the definition of ${\widetilde{\theta }}_\varrho$ (here, the right-hand side strictly decreases in the transition from ${\widetilde{\theta }}_\varrho$ to $\theta _-$, while the left-hand side increases, since $\Lambda _{\varrho }^{(\lambda , {\widetilde{\theta }}_\varrho )}(t_o) \subset \Lambda _{\varrho }^{(\lambda , \theta _-)}(t_o)$), and for *r* with $|r-\varrho |<\delta$ small enough the claim follows from the continuity of both sides as a function of *r*. The preceding inequality implies that ${\widetilde{\theta }}_r\ge \theta _-{:}{=}{\widetilde{\theta }}_\varrho -\varepsilon$. This completes the proof of the continuity of $(0,R_o]\ni \varrho \mapsto {\widetilde{\theta }}_\varrho$.

Unfortunately, the mapping ${\widetilde{\theta }}_\varrho$ might not be monotone in $\varrho$. For this reason we modify ${\widetilde{\theta }}_\varrho$, so that the modification—denoted by $\theta _\varrho$—becomes monotone. More precisely, we define

$$\begin{aligned} \theta _\varrho \equiv \theta _{z_o;\varrho }^{(\lambda )}{:}{=} \max _{r\in [\varrho ,R_o]} {\widetilde{\theta }}_{z_o;r}^{(\lambda )}. \end{aligned}$$

We only use the abbreviation of $\theta _{z_o;\varrho }^{(\lambda )}$ by $\theta _\varrho$ in a context in which $z_o$ and $\lambda$ are fixed, so that no confusion is possible. By construction the mapping $(0,R_o]\ni \varrho \mapsto \theta _\varrho$ is continuous and monotonically decreasing. Moreover, we claim that for radii $\varrho \le s$, the cylinders $Q_{s}^{(\lambda ,\theta _\varrho )}(z_o)$ are $\theta$-sub-intrinsic with constant $C_\theta =1$, i.e.6.4In fact, the definition of $\theta _s$ and the monotonicity of $\theta _\varrho$ imply ${\widetilde{\theta }}_s\le \theta _{s}\le \theta _{\varrho }$, so that $Q_{s}^{(\lambda ,\theta _{\varrho })}(z_o)\subset Q_{s}^{(\lambda ,{\widetilde{\theta }}_{s})}(z_o)$. Therefore, we haveThis proves the claim (6.4). We now define

6.5 $$\begin{aligned} {\widetilde{\varrho }} {:}{=} \left\{ \begin{array}{cl} R_o, &{}{} \quad \text{ if } \theta _\varrho =\lambda , \\ \inf \big \{s\in [\varrho , R_o]: \theta _s={\widetilde{\theta }}_s \big \}, &{}{} \quad \text{ if } \theta _\varrho >\lambda . \end{array} \right. \end{aligned}$$

In particular, for any $\sigma \in [\varrho ,{\widetilde{\varrho }}]$ we have $\theta _\sigma ={\widetilde{\theta }}_{\tilde{\varrho }}$. Next, we claim that

6.6 $$\begin{aligned} \theta _\varrho \le \Big (\frac{s}{\varrho }\Big )^{\frac{n+p^\sharp +q+1}{p^\sharp +q-1}} \theta _{s} \quad \text{ for } \text{ any } s\in (\varrho ,R_o]. \end{aligned}$$

If $\theta _\varrho =\lambda$, then also $\theta _s=\lambda$, so that (6.6) trivially follows. Therefore, it remains to consider the case $\theta _\varrho >\lambda$. Then, for any $s\in [{\widetilde{\varrho }},R_o]$ the monotonicity of $s\mapsto \theta _s$, (6.2) and (6.4) imply

$$\begin{aligned} \theta _\varrho ^{p^\sharp +q-1}&= {\widetilde{\theta }}_{{\widetilde{\varrho }}}^{p^\sharp +q-1} = \frac{\lambda ^{p-2}}{|Q_{{\widetilde{\varrho }}}|} \iint _{Q_{{\widetilde{\varrho }}}^{(\lambda ,\theta _{{\widetilde{\varrho }}})}(z_o)} \frac{|u|^{p^\sharp }}{{\widetilde{\varrho }}^{p^\sharp }} \,\mathrm {d}x\mathrm {d}t\\&\le \Big (\frac{s}{{\widetilde{\varrho }}}\Big )^{n+p^\sharp +q+1} \frac{\lambda ^{p-2}}{|Q_s|} \iint _{Q_{s}^{(\lambda ,\theta _s)}(z_o)} \frac{|u|^{p^\sharp }}{s^{p^\sharp }} \,\mathrm {d}x\mathrm {d}t\\&\le \Big (\frac{s}{\varrho }\Big )^{n+p^\sharp +q+1} \theta _{s}^{p^\sharp +q-1}, \end{aligned}$$

which yields (6.6). If $s\in (\varrho ,{\widetilde{\varrho }}]$, then $\theta _\varrho =\theta _{s}$, and the claim (6.6) follows again. Thus, we have established (6.6) in any case.

Next, we apply (6.6) with $s=R_o$. Since $\theta _{R_o}={\widetilde{\theta }}_{R_o}$, estimate (6.3) for ${\widetilde{\theta }}_{R_o}$ yields

6.7 $$\begin{aligned} \theta _\varrho \le \Big (\frac{R_o}{\varrho }\Big )^{\frac{n+p^\sharp +q+1}{p^\sharp +q-1}} \theta _{R_o} \le \Big (\frac{4R_o}{\varrho }\Big )^{\frac{n+p^\sharp +q+1}{p^\sharp +q-1}} \lambda . \end{aligned}$$

In the following, we consider the system of concentric cylinders $Q_{\varrho }^{(\lambda , \theta _{z_o;\varrho }^{(\lambda )})}(z_o)$ with radii $\varrho \in (0,R_o]$ and $z_o\in Q_{2R}$. The cylinders are nested in each other, in the sense that

$$\begin{aligned} Q_{r}^{(\lambda ,\theta _{z_o;r}^{(\lambda )})}(z_o) \subset Q_{s}^{(\lambda , \theta _{z_o;s}^{(\lambda )})}(z_o) \text{ whenever } 0<r<s\le R_o. \end{aligned}$$

The inclusion holds true due to the monotonicity of the mapping $\varrho \mapsto \theta _{z_o;\varrho }^{(\lambda )}$. The disadvantage of this system of nested cylinders is that in general the cylinders are not $\theta$-intrinsic, but merely $\theta$-sub-intrinsic with $C_\theta =1$.

### Covering property

Here, we will prove a *Vitali type covering lemma* for the cylinders constructed in the last section. More precisely, we will show the following.

#### Lemma 6.1

Let $\lambda \ge \lambda _o$. There exists a constant ${\hat{c}}={\hat{c}}(n,p,q)\ge 20$ such that the following holds true: Let ${\mathcal {F}}$ be any collection of cylinders $Q_{4r}^{(\lambda , \theta _{z;r}^{(\lambda )})}(z)$, where $Q_{r}^{(\lambda ,\theta _{z;r}^{(\lambda )})}(z)$ is a cylinder of the form as constructed in Sect. 6.1 with radius $r\in (0,\tfrac{R_o}{{\hat{c}}})$. Then, there exists a countable subfamily ${\mathcal {G}}$ of disjoint cylinders in ${\mathcal {F}}$ such that

6.8 $$\begin{aligned} \bigcup _{Q\in {\mathcal {F}}} Q \subset \bigcup _{Q\in {\mathcal {G}}} {\widehat{Q}}, \end{aligned}$$

where ${\widehat{Q}}$ denotes the $\frac{1}{4}{\hat{c}}$-times enlarged cylinder *Q*, i.e. if $Q=Q_{4r}^{(\lambda ,\theta _{z;r}^{(\lambda )})}(z)$, then $\widehat{Q}=Q_{{\hat{c}} r}^{(\lambda ,\theta _{z;r}^{(\lambda )})}(z)$.

#### Proof

Let ${\hat{c}}\ge 20$ be a parameter at our disposal. Later on we fix ${\hat{c}}$ in a universal way in dependence on *n*, *p*, and *q*. For $j\in {\mathbb {N}}$ we define

$$\begin{aligned} {\mathcal {F}}_j {:}{=} \big \{Q_{4r}^{(\lambda ,\theta _{z;r}^{(\lambda )})}(z)\in {\mathcal {F}}: \tfrac{R_o}{2^j{\hat{c}}}<r\le \tfrac{R_o}{2^{j-1}{\hat{c}}} \big \}. \end{aligned}$$

The subfamilies ${\mathcal {G}}_j\subset {\mathcal {F}}_j$ are constructed according to the following recursive scheme: Let ${\mathcal {G}}_1$ be any maximal disjoint collection of cylinders in ${\mathcal {F}}_1$. Note that ${\mathcal {G}}_1$ is finite, since by (6.7) and the definition of ${\mathcal {F}}_1$ the ${\mathcal {L}}^{n+1}$-measure of each cylinder $Q\in {\mathcal {G}}_1$ is uniformly bounded from below. Now, assume that ${\mathcal {G}}_1, {\mathcal {G}}_2, \dots , \mathcal G_{k-1}$ have already been inductively selected up to some $k\in {\mathbb {N}}_{\ge 2}$. Then, from ${\mathcal {F}}_k$ we remove those cylinders *Q* which intersect one of the previously chosen cylinders $Q^*\in {\mathcal {G}}_1\cup \ldots \cup {\mathcal {G}}_{k-1}$. Amongst the remaining cylinders we then choose a maximal disjoint sub-collection ${\mathcal {G}}_k$. In other words, we choose $\mathcal G_k$ as a maximal disjoint collection of cylinders in

$$\begin{aligned} \bigg \{Q\in {\mathcal {F}}_k: Q\cap Q^*=\emptyset \hbox { for any } \displaystyle Q^*\in \bigcup _{j=1}^{k-1} {\mathcal {G}}_j \bigg \}. \end{aligned}$$

Note again that ${\mathcal {G}}_k$ is finite. Finally, we define

$$\begin{aligned} {\mathcal {G}}{:}{=} \bigcup _{j=1}^\infty {\mathcal {G}}_j. \end{aligned}$$

By construction, ${\mathcal {G}}$ is a countable collection of disjoint cylinders and ${\mathcal {G}}\subset {\mathcal {F}}$.

At this point it remains to prove that for each $Q\in {\mathcal {F}}$ there exists a cylinder $Q^*\in {\mathcal {G}}$ such that $Q\cap Q^*\not =\emptyset$ and $Q\subset \widehat{Q}^*$. To this aim we choose some arbitrary $Q=Q_{4r}^{(\lambda ,\theta _{z;r}^{(\lambda )})}(z)\in {\mathcal {F}}$. Then, there exists an index $j\in {\mathbb {N}}$ such that $Q\in {\mathcal {F}}_j$. The maximality of ${\mathcal {G}}_j$ ensures the existence of some cylinder $Q_*=Q_{4r_*}^{(\lambda ,\theta _{z_*;r_*}^{(\lambda )})}(z_*)\in \bigcup _{i=1}^{j} {\mathcal {G}}_i$ with $Q\cap Q_*\not =\emptyset$. Observe that $r\le \tfrac{R_o}{2^{j-1}{\hat{c}}}$ and $r_*>\tfrac{R_o}{2^j{\hat{c}}}$, so that $r<2r_*$. In the following we shall establish an estimate relating the size of $\theta _{z_*;r_*}^{(\lambda )}$ to the one of $\theta _{z;r}^{(\lambda )}$. The precise estimate is as follows:

6.9 $$\begin{aligned} \theta _{z_*;r_*}^{(\lambda )} \le 64^{\frac{n+p^\sharp +q+1}{p^\sharp +q-1}}\, \theta _{z;r}^{(\lambda )}\,. \end{aligned}$$

Let $\eta {:}{=}16$. Denote by ${\widetilde{r}}_*\in [r_*,R_o]$ the radius associated to the cylinder $Q_{r_*}^{(\lambda ,\theta _{z_*;r_*}^{(\lambda )})}(z_*)$; see (6.5) for the construction. Recall that either $Q_{\widetilde{r}_*}^{(\lambda ,\theta _{z_*;r_*}^{(\lambda )})}(z_*)$ is intrinsic in the sense of (6.2)$_2$ or $\widetilde{r}_*=R_o$ and $\theta _{z_*;r_*}^{(\lambda )}=\lambda$. In the latter case we have

$$\begin{aligned} \theta _{z_*;r_*}^{(\lambda )} = \lambda \le \theta _{z;r}^{(\lambda )}, \end{aligned}$$

by definition of $\theta _{z;r}^{(\lambda )}$, so that the claim (6.9) is clearly satisfied. Therefore, it only remains to consider the case that $Q_{\widetilde{r}_*}^{(\lambda ,\theta _{z_*;r_*}^{(\lambda )})}(z_*)$ is intrinsic, more precisely

6.10 $$\begin{aligned} (\theta _{z_*;r_*}^{(\lambda )})^{p^\sharp +q-1} = \frac{\lambda ^{p-2}}{|Q_{{\widetilde{r}}_*}|} \iint _{Q_{\widetilde{r}_*}^{(\lambda ,\theta _{z_*;r_*}^{(\lambda )})}(z_*)} \frac{|u|^{p^\sharp }}{{\widetilde{r}}_*^{p^\sharp }} \,\mathrm {d}x\mathrm {d}t. \end{aligned}$$

Now, we distinguish between the cases ${\widetilde{r}}_*\le \frac{R_o}{\eta }$ and ${\widetilde{r}}_*> \frac{R_o}{\eta }$. In the latter case we exploit (6.10), the definition of $\lambda _o$ and the facts $\theta _{z;r}^{(\lambda )}\ge \lambda \ge \lambda _o$ in order to obtain

$$\begin{aligned} \big (\theta _{z_*;r_*}^{(\lambda )}\big )^{p^\sharp +q-1}&\le \Big (\frac{4R}{{\widetilde{r}}_*}\Big )^{n+p^\sharp +q+1} \frac{\lambda ^{p-2}}{|Q_{R}|} \iint _{Q_{4R}} \frac{|u|^{p^\sharp }}{(4R)^{p^\sharp }} \,\mathrm {d}x\mathrm {d}t\\&\le (4\eta )^{n+p^\sharp +q+1}\Big (\frac{R}{R_o}\Big )^{n+p^\sharp +q+1} \lambda ^{p-2}\lambda _o^{\frac{p}{d}} \\&\le (4\eta )^{n+p^\sharp +q+1} \big [\theta _{z;r}^{(\lambda )}\big ]^{p^\sharp +q-1}, \end{aligned}$$

where the last estimate follows by distinguishing between the cases $p\ge q+1$ and $p<q+1$. The preceding inequality shows that

$$\begin{aligned} \theta _{z_*;r_*}^{(\lambda )} \le (4\eta )^{\frac{n+p^\sharp +q+1}{p^\sharp +q-1}}\theta _{z;r}^{(\lambda )}, \end{aligned}$$

proving (6.9) in the case $\widetilde{r}_*>\frac{R}{\eta }$. It remains to consider the case $\widetilde{r}_*\le \frac{R_o}{\eta }$. Since ${\tilde{r}}_*\ge r_*$ and $|x-x_*|<4r+4r_*\le 12r_*$, we know $B_{4{\tilde{r}}_*}(x_*)\subset B_{16{\tilde{r}}_*}(x)= B_{\eta \tilde{r}_*}(x)$. Moreover,

6.11 $$\begin{aligned} |t-t_*| \le \big (\theta _{z;r}^{(\lambda )}\big )^{q-1}\lambda ^{2-p}(4r)^{1+q} + \big (\theta _{z_*;r_*}^{(\lambda )}\big )^{q-1}\lambda ^{2-p}(4r_*)^{1+q}. \end{aligned}$$

At this point, we can assume $\theta _{z;r}^{(\lambda )}\le \theta _{z_*;r_*}^{(\lambda )}$. Otherwise (6.9) trivially holds. The monotonicity of $\varrho \mapsto \theta _{z;\varrho }^{(\lambda )}$ and the fact that $r\le 2r_*\le 2 {\widetilde{r}}_*\le \eta {\widetilde{r}}_*$ implies that

6.12 $$\begin{aligned} \theta _{z_*;r_*}^{(\lambda )} \ge \theta _{z;r}^{(\lambda )} \ge \theta _{z;\eta {\widetilde{r}}_*}^{(\lambda )}. \end{aligned}$$

Therefore, we can conclude that

$$\begin{aligned} \big (\theta _{z_*;r_*}^{(\lambda )}\big )^{q-1}&\lambda ^{2-p}(4\widetilde{r}_*)^{1+q}+ |t-t_*|\\&\le 2\big (\theta _{z_*;r_*}^{(\lambda )}\big )^{q-1}\lambda ^{2-p}(4{\widetilde{r}}_*)^{1+q} + \big (\theta _{z;r}^{(\lambda )}\big )^{q-1}\lambda ^{2-p}(4r)^{1+q}\\&\le 2^{q+2} \big (\theta _{z;\eta {\widetilde{r}}_*}^{(\lambda )}\big )^{q-1}\lambda ^{2-p}(4{\widetilde{r}}_*)^{1+q}\\&\le \big (\theta _{z;\eta {\widetilde{r}}_*}^{(\lambda )}\big )^{q-1}\lambda ^{2-p}\big (\eta {\widetilde{r}}_*\big )^{1+q}, \end{aligned}$$

and this immediately implies the inclusion

$$\begin{aligned} \Lambda _{4{\widetilde{r}}_*}^{(\lambda ,\theta _{z_*;r_*}^{(\lambda )})}(t_*) \subset \Lambda _{\eta {\widetilde{r}}_*}^{(\lambda ,\theta _{z;\eta {\tilde{r}}_*}^{(\lambda )})}(t). \end{aligned}$$

Altogether, we have shown

$$\begin{aligned} Q_{4{\widetilde{r}}_*}^{(\lambda ,\theta _{z_*;r_*}^{(\lambda )})}(z_*) \subset Q_{\eta {\widetilde{r}}_*}^{(\lambda ,\theta _{z;\eta {\tilde{r}}_*}^{(\lambda )})}(z). \end{aligned}$$

Using (6.10), the inequalities (6.12), and (6.4) with $\varrho =s=\eta {\tilde{r}}_*$, we obtain

$$\begin{aligned} \big (\theta _{z_*;r_*}^{(\lambda )}\big )^{p^\sharp +q-1}&\le \frac{\eta ^{p^\sharp }\lambda ^{p-2}}{|Q_{{\widetilde{r}}_*}|} \iint _{Q_{\eta {\widetilde{r}}_*}^{(\lambda ,\theta _{z;\eta {\widetilde{r}}_*}^{(\lambda )})}(z)} \frac{|u|^{p^\sharp }}{(\eta {\widetilde{r}}_*)^{p^\sharp }} \,\mathrm {d}x\mathrm {d}t\le \eta ^{n+p^\sharp +q+1} (\theta _{z;r}^{(\lambda )})^{p^\sharp +q-1} . \end{aligned}$$

We conclude

$$\begin{aligned} \theta _{z_*;r_*}^{(\lambda )} \le \eta ^{\frac{n+p^\sharp +q+1}{p^\sharp +q-1}}\,\theta _{z;r}^{(\lambda )}, \end{aligned}$$

so that (6.9) is established in all cases. From (6.9) we get

$$\begin{aligned} \lambda ^{2-p}&\big (\theta _{z;r}^{(\lambda )}\big )^{q-1}(4r)^{1+q} + |t-t_*|\\&\le 2\lambda ^{2-p}\big (\theta _{z;r}^{(\lambda )}\big )^{q-1}(4r)^{1+q} + \lambda ^{2-p}\big (\theta _{z_*;r_*}^{(\lambda )}\big )^{q-1}(4r_*)^{1+q}\\&\le \Big (2\cdot 64^{\frac{n+p^\sharp +q+1}{p^\sharp +q-1}(1-q)}+1\Big ) \lambda ^{2-p}\big (\theta _{z_*;r_*}^{(\lambda )}\big )^{q-1}(4r_*)^{1+q} \\&\le \lambda ^{2-p}\big (\theta _{z_*;r_*}^{(\lambda )}\big )^{q-1} ({\hat{c}} r_*)^{1+q}, \end{aligned}$$

if we choose the constant ${\hat{c}}={\hat{c}}(n,p,q)\ge 20$ suitably. Moreover, we note that

$$\begin{aligned} 4r+|x-x_*|\le 8r+4r_*\le 20r_*\le {\hat{c}}r_*. \end{aligned}$$

Therefore, we have shown that

$$\begin{aligned} Q =Q_{4r}^{(\lambda ,\theta _{z;r}^{(\lambda )})}(z)\ \subset {\widehat{Q}}^*\equiv Q_{{\hat{c}} r_*}^{(\lambda ,\theta _{z_*;r_*}^{(\lambda )})}(z_*). \end{aligned}$$

This proves the claim (6.8) and completes the proof. $\square$

### Stopping time argument

We now let6.13Obviously, this condition implies the previously demanded requirement (6.1). For $\lambda >\lambda _o$ and $r\in (0,2R]$, we define the superlevel set of |*Du*| by

$$\begin{aligned} {{\textbf {E}}}(r,\lambda ):= \Big \{z\in Q_{r}: z \text { is a Lebesgue point of }|Du| \text { and } |Du|(z) > \lambda \Big \}. \end{aligned}$$

In this context, of course, the concept of Lebesgue points of |*Du*| must be adapted to the system of cylinders constructed in Sect. 6.1. For radii $R\le R_1<R_2\le 2R$, we consider the concentric parabolic cylinders $Q_R\subseteq Q_{R_1} \subset Q_{R_2}\subseteq Q_{2R}$. Note that the inclusion $Q_{2\varrho }^{(\lambda ,\theta )}(z_o) \subset Q_{4R}$ holds true, whenever $z_o\in Q_{2R}$, $\theta \ge \lambda$ and $\varrho \in (0,R_o]$. We fix $z_o\in {\varvec{E}}(R_1,\lambda )$ and abbreviate $\theta _s\equiv \theta _{z_o;s}^{(\lambda )}$ for $s\in (0,R_o]$ throughout this section. By definition of $\varvec{E}(R_1,\lambda )$, we have that6.14In the following, we consider values of $\lambda$ satisfying

6.15 $$\begin{aligned} \lambda> B\lambda _o, \qquad \hbox {where } \quad B {:}{=} \Big (\frac{4{\hat{c}} R}{R_2-R_1}\Big )^{\frac{dp^\sharp (n+2)}{p(p^\sharp +q-1)}} >1, \end{aligned}$$

where ${\hat{c}}={\hat{c}}(n,p,q)$ denotes the constant from the Vitali-type covering Lemma 6.1. For *s* with

6.16 $$\begin{aligned} \frac{R_2-R_1}{\varvec{\mathfrak {m}}} \le s \le R_o \qquad \hbox {with }\quad \varvec{\mathfrak {m}}:=\frac{{\hat{c}}}{\min \{1,\lambda ^{\frac{p-q-1}{q+1}}\}} \end{aligned}$$

we have, by definition of $\lambda _o$, thatIn the individual intermediate steps we have used in turn (6.7), (6.16) and (6.15), and distinguished between the cases $p\ge q+1$ and $p<q+1$. On the other hand, on behalf of (6.14) we find $0< s < \tfrac{R_2-R_1}{\varvec{\mathfrak {m}}}$ sufficiently small such that the integral in (6.14) with $Q_{s}^{(\lambda ,\theta _s)}(z_o)$ as domain of integration, possesses a value larger than $\lambda ^{p}$. Consequently, by the absolute continuity of the integral there exists a maximal radius $0<\varrho _{z_o} < \tfrac{R_2-R_1}{\varvec{\mathfrak {m}}}$ such that6.17The choice of $\varrho _{z_o}$ as maximal radius now guarantees6.18More generally, for any $\varrho _{z_o}<s\le R_o$, the monotonicity of $\varrho \mapsto \theta _\varrho$, estimate (6.6) and (6.18) imply6.19Moreover, $Q_{{\hat{c}}\varrho _{z_o}}^{(\lambda ,\theta _{\varrho _{z_o}})}(z_o)\subset Q_{R_2}$. In fact, this inclusion follows from the fact $\varrho _{z_o}<\frac{R_2-R_1}{\varvec{\mathfrak {m}}}$ since this implies

$$\begin{aligned} R_1^{q+1}+\lambda ^{2-p}\theta _{\varrho _{z_o}}^{q-1}\min \{1,\lambda ^{p-q-1}\}(R_2-R_1)^{q+1} \le R_1^{q+1}+(R_2-R_1)^{q+1} \le R_2^{q+1}. \end{aligned}$$

### A Reverse–Hölder inequality

As before, we consider $z_o\in {\varvec{E}}(R_1,\lambda )$ with $\lambda$ as in (6.15). Since $\lambda$ and $z_o$ are fixed, it is possible to use the abbreviation $\theta _{\varrho _{z_o}} {:}{=} \theta _{z_o;\varrho _{z_o}}^{(\lambda )}$ without causing confusion. We keep in mind that by construction $0<\varrho _{z_o}< \frac{R_2-R_1}{\varvec{\mathfrak {m}}}$. We choose ${\widetilde{\varrho }}_{z_o}\in [\varrho _{z_o},R]$ according to (6.5). This choice allows us to pass from the possibly sub-intrinsic cylinder $Q_{\varrho _{z_o}}^{(\lambda ,\theta _{\varrho _{z_o}})}(z_o)$ to the intrinsic cylinder $Q_{{\widetilde{\varrho }}_{z_o}}^{(\lambda ,\theta _{\varrho _{z_o}})}(z_o)$. By construction we have $\theta _s=\theta _{\varrho _{z_o}}$ for any $s\in [\varrho _{z_o}, {\widetilde{\varrho }}_{z_o}]$ and in particular, $\theta _{{\widetilde{\varrho }}_{z_o}}=\theta _{\varrho _{z_o}}$. Our aim now is to prove the following reverse Hölder inequality6.20with $\vartheta {:}{=}\max \big \{\frac{n(q+1)}{p(n+q+1)},\frac{n}{n+q+1},\frac{1}{p}, \frac{p-1}{p}\big \}<1$ and $c=c(n,p,q,\nu , L)$. We distinguish between the two cases in which ${\widetilde{\varrho }}_{z_o}\le 2\varrho _{z_o}$ or ${\widetilde{\varrho }}_{z_o}> 2\varrho _{z_o}$.

**The case **$\varvec{{\widetilde{\varrho }}_{z_o}\le 2\varrho _{z_o}}$. Here we wish to apply Proposition 5.1 on the cylinder $Q_{{\widetilde{\varrho }}_{z_o}}^{(\lambda ,\theta _{\varrho _{z_o}})}(z_o)$. In order to check that this is possible, we first note that $\theta _{\varrho _{z_o}}>\lambda$, since otherwise we would have ${\widetilde{\varrho }}_{z_o}=R_o>2\varrho _{z_o}$. Therefore, we can apply (6.2). Keeping in mind that $\theta _{\varrho _{z_o}}=\theta _{\tilde{\varrho }_{z_o}}=\tilde{\theta }_{\tilde{\varrho }_{z_o}}$ and using additionally (6.4) with $s=2{\widetilde{\varrho }}_{z_o}$, we deduceThis shows that $Q_{{\widetilde{\varrho }}_{z_o}}^{(\lambda ,\theta _{\varrho _{z_o}})}(z_o)$ is $\theta$-intrinsic. More precisely, the coupling condition (4.2) is satisfied with constant $C_\theta =1$. On the other hand, estimate (6.19) with $s=2{\widetilde{\varrho }}_{z_o}\le 4\varrho _{z_o}$ and the identity (6.17) implywith a constant $c=c(n,p,q)$. This ensures that the $\lambda$-intrinsic coupling condition (4.1) is satisfied for the cylinder $Q_{{\widetilde{\varrho }}_{z_o}}^{(\lambda ,\theta _{\varrho _{z_o}})}(z_o)$, with a constant $C_\lambda$ depending on *n*, *p*,  and *q*. Altogether, we have shown that the assumptions of Proposition 5.1 are satisfied. Applying this proposition and using the assumption ${\widetilde{\varrho }}_{z_o}\le 2\varrho _{z_o}$, we infer the reverse Hölder inequalitywhere $c=c(n,p,q,\nu ,L)$. This establishes (6.20) in the first case.

**The case** $\varvec{{\widetilde{\varrho }}_{z_o}> 2\varrho _{z_o}}$. Here, we will apply Proposition 5.2 on the cylinder $Q_{\varrho _{z_o}}^{(\theta _{\varrho _{z_o}})}(z_o)$. This is only permitted, however, if the hypotheses (4.1) and (5.6) are satisfied on this cylinder. First, we notice that (4.1) (with $C_\lambda =1$) follows from (6.17) and (6.18). Moreover, the condition (5.6)$_1$ (with $C_\theta \equiv 1$) is an immediate consequence of (6.4). For the verification of (5.6)$_2$, we consider two cases. In the case ${\widetilde{\varrho }}_{z_o}\in (\frac{R_o}{2},R_o]$, we use estimate (6.7) with $\varrho ={\widetilde{\varrho }}_{z_o}$ and then (6.17) to deducewith $c=c(n,p,q)$, which yields (5.6)$_2$ in the first case. In the second case ${\widetilde{\varrho }}_{z_o}\in (2\varrho _{z_o},\frac{R_o}{2}]$, we observe that by (6.2) with $\varrho ={\widetilde{\varrho }}_{z_o}$ and by (6.4) with $s=2{\widetilde{\varrho }}_{z_o}$, the cylinder $Q_{{\widetilde{\varrho }}_{z_o}}^{(\lambda ,\theta _{\varrho _{z_o}})}(z_o)$ satisfies the $\theta$-intrinsic coupling condition (4.2) with constant $C_\theta =1$. Because of (6.17), applied with ${\widetilde{\varrho }}_{z_o}$ in place of $\varrho _{z_o}$, the cylinder $Q_{{\widetilde{\varrho }}_{z_o}}^{(\lambda ,\theta _{\varrho _{z_o}})}(z_o)$ is $\lambda$-intrinsic as well. Because of $2{\widetilde{\varrho }}_{z_o}\le R_o$, the cylinder with doubled radius is still contained in $Q_{4R}\subset \Omega _T$. Therefore, the assumptions of Lemma 4.6 are satisfied on $Q_{{\widetilde{\varrho }}_{z_o}}^{(\lambda ,\theta _{\varrho _{z_o}})}(z_o)$, and we infer the estimatewith $c=c(n,p,q,\nu ,L)$, where the last estimate holds since ${\widetilde{\varrho }}_{z_o}>2\varrho _{z_o}$ ensures that (6.4) is applicable with $s={\widetilde{\varrho }}_{z_o}/2$. The last term can be absorbed into the left-hand side, and we conclude that the boundis satisfied in all cases, with a constant $c=c(n,p,q,\nu ,L)$. The last identity is due to (6.17). This proves that (5.6)$_2$ is satisfied, and we have verified all assumptions of Proposition 5.2 for the cylinder $Q_{\varrho _{z_o}}^{(\theta _{\varrho _{z_o}})}(z_o)$. Therefore, we obtain the reverse Hölder inequalityOverall, we have shown that in any case the reverse Hölder inequality (6.20) holds.

### Estimate on super-level sets

Up to this point, we have shown: If $$\lambda $$ satisfies (6.15), then for every $$z_o\in E(R_1,\lambda )$$ there exists a cylinder $$Q_{\varrho _{z_o}}^{(\lambda ,\theta _{z_o;\varrho _{z_o}}^{(\lambda )})}(z_o)$$ such that the $${\hat{c}}$$ times enlarged cylinder $$Q_{{\hat{c}}\varrho _{z_o}}^{(\lambda ,\theta _{z_o;\varrho _{z_o}}^{(\lambda )})}(z_o)$$ is still contained in $$ Q_{R_2}$$, and such that (6.17), (6.18) and (6.20) hold on this specific cylinder. As before, we write $$\theta _{\varrho _{z_o}}\equiv \theta _{z_o;\varrho _{z_o}}^{(\lambda )}$$. Next we define the superlevel set of the inhomogeneity |*F*| by

$$\begin{aligned} {{{\textbf {F}}}}(r,\lambda ){:}{=} \Big \{z\in Q_{r}: z \text { is a Lebesgue point of } |F| \text { and } |F|(z)>\lambda \Big \}.\end{aligned}$$

Using (6.17) and (6.20) we obtain, for $\eta \in (0,1]$ to be specified later, thatfor a constant $c=c(n,p,q,\nu ,L)$. In the preceding term we used the abbreviationFor the estimation of $\mathrm I$ we use Hölder’s inequality and (6.19). This leads towith $c=c(n,p,q)$. We insert this above and choose $\eta =(\frac{1}{2c})^{\frac{1}{p}}$. This allows to re-absorb the resulting term $\frac{1}{2}\lambda ^{p}$ into the left-hand side. Then, we multiply the result by $\big |Q_{4\varrho _{z_o}}^{(\lambda ,\theta _{\varrho _{z_o}})}(z_o)\big |$. This leads to the inequality

$$\begin{aligned} \lambda ^{p}\big |Q_{4\varrho _{z_o}}^{(\lambda ,\theta _{\varrho _{z_o}})}(z_o)\big |&\le c\iint _{Q_{4\varrho _{z_o}}^{(\lambda ,\theta _{\varrho _{z_o}})}(z_o)\cap {\mathbf {E}}(R_2,\eta \lambda )} \lambda ^{(1-\vartheta )p}|Du|^{\vartheta p} \,\mathrm {d}x\mathrm {d}t\\&\quad + c\, \iint _{Q_{4\varrho _{z_o}}^{(\lambda ,\theta _{\varrho _{z_o}})}(z_o)\cap {\mathbf {F}}(R_2,\eta \lambda )} |F|^{p} \,\mathrm {d}x\mathrm {d}t, \end{aligned}$$

again with $c=c(n,p,q,\nu ,L)$. Now, (6.19) for the choice $s={\hat{c}}\varrho _{z_o}$ allows us to estimate $\lambda ^{p}$ from below byInserting this above and keeping in mind that ${\hat{c}}$ depends only on *n*, *p*, and *q*, we deduce

6.21 $$\begin{aligned} \iint _{Q_{{\hat{c}}\varrho _{z_o}}^{(\lambda ,\theta _{\varrho _{z_o}})}(z_o)} |Du|^p \,\mathrm {d}x\mathrm {d}t&\le c \iint _{Q_{4\varrho _{z_o}}^{(\lambda ,\theta _{\varrho _{z_o}})}(z_o)\cap {\varvec{E}}(R_2,\eta \lambda )} \lambda ^{(1-\vartheta )p}|Du|^{\vartheta p} \,\mathrm {d}x\mathrm {d}t\nonumber \\&\quad + c \iint _{Q_{4\varrho _{z_o}}^{(\lambda ,\theta _{\varrho _{z_o}})}(z_o)\cap {\varvec{F}}(R_2,\eta \lambda )} |F|^{p} \,\mathrm {d}x\mathrm {d}t. \end{aligned}$$

To summarize: so far we have shown that for any value $\lambda >B\lambda _o$ the associated super-level set $\varvec{E}(R_1,\lambda )$ is covered by a family

$$\begin{aligned} \mathcal F\equiv \Big \{Q_{4\varrho _{z_o}}^{(\lambda ,\theta _{z_o;\varrho _{z_o}}^{(\lambda )})}(z_o)\Big \} \end{aligned}$$

of parabolic cylinders with center $z_o\in \varvec{E}(R_1,\lambda )$ which are contained in $Q_{R_2}$, and such that (6.21) holds true on each of these cylinders. At this point, we use the Vitali type covering property from Sect.  6.2 and obtain a countable subfamily

$$\begin{aligned} \Big \{Q_{4\varrho _{z_i}}^{(\lambda ,\theta _{z_i;\varrho _{z_i}}^{(\lambda )})}(z_i)\Big \}_{i\in {\mathbb {N}}} \subset {\mathcal {F}} \end{aligned}$$

consisting of pairwise disjoint cylinders, such that the $\frac{1}{4}{\hat{c}}$-times enlarged cylinders $Q_{{\hat{c}}\varrho _{z_i}}^{(\lambda ,\theta _{z_i;\varrho _{z_i}}^{(\lambda )})}(z_i)$ cover the super-level set ${\varvec{E}}(R_1,\lambda )$ and are still contained in $Q_{R_2}$, i.e.  

$$\begin{aligned} {\varvec{E}}(R_1,\lambda ) \subset \bigcup _{i=1}^\infty Q_{{\hat{c}}\varrho _{z_i}}^{(\lambda ,\theta _{z_i;\varrho _{z_i}}^{(\lambda )})}(z_i) \subset Q_{R_2}. \end{aligned}$$

Since the $Q_{4\varrho _{z_i}}^{(\lambda ,\theta _{z_i;\varrho _{z_i}}^{(\lambda )})}(z_i)$ are pairwise disjoint we obtain with (6.21) that

$$\begin{aligned} \iint _{{\varvec{E}}(R_1,\lambda )}&|Du|^p \,\mathrm {d}x\mathrm {d}t\\&\le \sum _{i=1}^\infty \iint _{Q_{{\hat{c}}\varrho _{z_i}}^{(\lambda ,\theta _{z_i;\varrho _{z_i}}^{(\lambda )})}(z_i)} |Du|^p \,\mathrm {d}x\mathrm {d}t\\&\le c\sum _{i=1}^\infty \iint _{Q_{4\varrho _{z_i}}^{(\lambda ,\theta _{z_i;\varrho _{z_i}}^{(\lambda )})}(z_i)\cap {\varvec{E}}(R_2,\eta \lambda )} \lambda ^{(1-\vartheta )p}|Du|^{\vartheta p} \,\mathrm {d}x\mathrm {d}t\\&\quad + c\sum _{i=1}^\infty \iint _{Q_{4\varrho _{z_i}}^{(\lambda ,\theta _{z_i;\varrho _{z_i}}^{(\lambda )})}(z_i)\cap {\varvec{F}}(R_2,\eta \lambda )} |F|^{p} \,\mathrm {d}x\mathrm {d}t\\&\le c\iint _{{\varvec{E}}(R_2,\eta \lambda )} \lambda ^{(1-\vartheta )p} |Du|^{\vartheta p} \,\mathrm {d}x\mathrm {d}t+ c\iint _{{\varvec{F}}(R_2,\eta \lambda )} |F|^{p} \,\mathrm {d}x\mathrm {d}t, \end{aligned}$$

for a constant $c=c(n, p, q, \nu , L)$. On $\varvec{E}(R_1,\eta \lambda )\setminus {\varvec{E}}(R_1,\lambda )$ we have $|Du|\le \lambda$ and therefore

$$\begin{aligned} \iint _{{\varvec{E}}(R_1,\eta \lambda )\setminus {\mathbf {E}}(R_1,\lambda )} |Du|^p \,\mathrm {d}x\mathrm {d}t&\le \iint _{{\varvec{E}}(R_1,\eta \lambda )\setminus {\varvec{E}}(R_1,\lambda )} \lambda ^{(1-\vartheta )p}|Du|^{\vartheta p} \,\mathrm {d}x\mathrm {d}t\\&\le \iint _{{\varvec{E}}(R_2,\eta \lambda )} \lambda ^{(1-\vartheta )p}|Du|^{\vartheta p} \,\mathrm {d}x\mathrm {d}t. \end{aligned}$$

We add the two preceding inequalities and obtain

$$\begin{aligned} \iint _{{\varvec{E}}(R_1,\eta \lambda )} |Du|^p \,\mathrm {d}x\mathrm {d}t\le c\iint _{{\varvec{E}}(R_2,\eta \lambda )} \lambda ^{(1-\vartheta )p}|Du|^{\vartheta p} \,\mathrm {d}x\mathrm {d}t+ c \iint _{{\varvec{F}}(R_2,\eta \lambda )} |F|^p \,\mathrm {d}x\mathrm {d}t. \end{aligned}$$

In this inequality we replace $\eta \lambda$ by $\lambda$ and recall that $\eta =\eta (n,p,q,\nu ,L)<1$. With this replacement we obtain for any $\lambda \ge \eta B\lambda _o{=}{:}\lambda _1$ that

6.22 $$\begin{aligned} \iint _{{\varvec{E}}(R_1,\lambda )}&|Du|^p \,\mathrm {d}x\mathrm {d}t\nonumber \\&\le c \iint _{{\varvec{E}}(R_2,\lambda )} \Big (\frac{\lambda }{\eta }\Big )^{(1-\vartheta )p} |Du|^{\vartheta p} \,\mathrm {d}x\mathrm {d}t+ c \iint _{{\varvec{F}}(R_2,\lambda )} |F|^{p} \,\mathrm {d}x\mathrm {d}t\nonumber \\&\le c\iint _{{\varvec{E}}(R_2,\lambda )} \lambda ^{(1-\vartheta )p}|Du|^{\vartheta p} \,\mathrm {d}x\mathrm {d}t+ c \iint _{{\varvec{F}}(R_2,\lambda )} |F|^{p} \,\mathrm {d}x\mathrm {d}t\end{aligned}$$

holds true with a constant $c=c(n,p,q,\nu ,L)$. With this we have derived the desired *reverse Hölder inequality on super-level sets*.

### Proof of the gradient estimate

In principle, the quantitative higher integrability estimate would follow from the reverse Hölder inequality on super-level sets by multiplying (6.22) by $\lambda ^{\varepsilon p-1}$ and then integrating with respect to $\lambda$. This procedure would lead to an integral of $|Du|^{p(1+\varepsilon )}$ over $Q_{R_1}$ on the left-hand side, while on the right-hand side the same integral appears with $Q_{R_2}$ as domain of integration. However, it is not clear in advance that these integrals are finite for $R\le R_1<R_2\le 2R$. For this reason one must avoid powers of |*Du*| that are larger than *p*. This can be achieved by a truncation argument. The rigorous argument is as follows: For $k> \lambda _1$ the *truncation* of |*Du*| is defined by

$$\begin{aligned} |Du|_k:= \min \big \{|Du|, k\big \}, \end{aligned}$$

and for $r\in (0,2R]$ the corresponding super-level set by

$$\begin{aligned} {\varvec{E}}_k(r,\lambda ):= \big \{z\in Q_r: |Du|_k>\lambda \big \}. \end{aligned}$$

With these definitions, we infer the following version of (6.22) for the truncated gradient.

6.23 $$\begin{aligned}&\iint _{{\varvec{E}}_k(R_1,\lambda )} |Du|_k^{p(1-\vartheta )}|Du|^{\vartheta p} \,\mathrm {d}x\mathrm {d}t\nonumber \\&\qquad \le c\iint _{{\varvec{E}}_k(R_2,\lambda )} \lambda ^{(1-\vartheta )p}|Du|^{\vartheta p} \,\mathrm {d}x\mathrm {d}t+ c \iint _{{\varvec{F}}(R_2,\lambda )} |F|^{p} \,\mathrm {d}x\mathrm {d}t. \end{aligned}$$

This inequality is obviously true for values $k\le \lambda$, since then ${\varvec{E}}_k(r,\lambda )=\emptyset$, while for values $k>\lambda$ the claim immediately follows from (6.22), since in this case ${\varvec{E}}_k(r,\lambda )={\varvec{E}}(r,\lambda )$, and $|Du|_k\le |Du|$ a.e. Now, we multiply (6.23) by $\lambda ^{\varepsilon p-1}$, with a parameter $\epsilon \in (0,1]$ that will be chosen later in a universal way. We integrate the result with respect to $\lambda$ over the interval $(\lambda _1,\infty )$ and obtain

$$\begin{aligned} \int _{\lambda _1}^\infty \lambda ^{\varepsilon p-1}&\bigg [\iint _{{\varvec{E}}_k(R_1,\lambda )} |Du|_k^{(1-\vartheta )p} |Du|^{\vartheta p} \,\mathrm {d}x\mathrm {d}t\bigg ]\text {d}\lambda \\&\le c\int _{\lambda _1}^\infty \lambda ^{(1-\vartheta +\varepsilon )p-1} \bigg [\iint _{{\varvec{E}}_k(R_2,\lambda )} |Du|^{\vartheta p} \,\mathrm {d}x\mathrm {d}t\bigg ]\text {d}\lambda \\&\quad + c \int _{\lambda _1}^\infty \lambda ^{\varepsilon p-1} \bigg [\iint _{{\varvec{F}}(R_2,\lambda )} |F|^{p} \,\mathrm {d}x\mathrm {d}t\bigg ]\text {d}\lambda . \end{aligned}$$

Here, we exchange the order of integration in the individual integrals by an application of Fubini’s theorem. For the integral on the left-hand side, Fubini’s theorem implies

$$\begin{aligned} \int _{\lambda _1}^\infty&\lambda ^{\varepsilon p-1} \iint _{{\varvec{E}}_k(R_1,\lambda )} |Du|_k^{(1-\vartheta )p}|Du|^{\vartheta p} \,\mathrm {d}x\mathrm {d}t\text {d}\lambda \\&= \iint _{{\varvec{E}}_k(R_1,\lambda _1)} |Du|_k^{(1-\vartheta )p}|Du|^{\vartheta p} \int _{\lambda _1}^{|Du|_k} \lambda ^{\varepsilon p-1} \text {d}\lambda \,\mathrm {d}x\mathrm {d}t\\&= \tfrac{1}{\varepsilon p} \iint _{{\varvec{E}}_k(R_1,\lambda _1)} \Big [|Du|_k^{(1-\vartheta +\varepsilon )p}|Du|^{\vartheta p} - \lambda _1^{\varepsilon p} |Du|_k^{(1-\vartheta )p}|Du|^{\vartheta p} \Big ] \,\mathrm {d}x\mathrm {d}t, \end{aligned}$$

while for the first integral on the right-hand side we find that

$$\begin{aligned} \int _{\lambda _1}^\infty&\lambda ^{(1-\vartheta +\varepsilon )p-1} \iint _{{\varvec{E}}_k(R_2,\lambda )} |Du|^{\vartheta p} \,\mathrm {d}x\mathrm {d}t\text {d}\lambda \\&= \iint _{{\varvec{E}}_k(R_2,\lambda _1)} |Du|^{\vartheta p} \int _{\lambda _1}^{|Du|_k} \lambda ^{(1-\vartheta +\varepsilon )p-1} \text {d}\lambda \,\mathrm {d}x\mathrm {d}t\\&\le \tfrac{1}{(1-\vartheta +\varepsilon )p} \iint _{{\varvec{E}}_k(R_2,\lambda _1)} |Du|_k^{(1-\vartheta +\varepsilon )p} |Du|^{\vartheta p}\,\mathrm {d}x\mathrm {d}t\\&\le \tfrac{1}{(1-\vartheta )p} \iint _{{\varvec{E}}_k(R_2,\lambda _1)} |Du|_k^{(1-\vartheta +\varepsilon )p} |Du|^{\vartheta p}\,\mathrm {d}x\mathrm {d}t. \end{aligned}$$

Finally, for the second integral on the right-hand side we obtain

$$\begin{aligned} \int _{\lambda _1}^\infty \lambda ^{\varepsilon p-1} \iint _{{\varvec{F}}(R_2,\lambda )} |F|^{p} \,\mathrm {d}x\mathrm {d}t\text {d}\lambda&= \iint _{{\varvec{F}}(R_2,\lambda _1)} |F|^{p} \int _{\lambda _1}^{|F|} \lambda ^{\varepsilon p-1} \text {d}\lambda \,\mathrm {d}x\mathrm {d}t\\&\le \tfrac{1}{\varepsilon p} \iint _{{\varvec{F}}(R_2,\lambda _1)} |F|^{(1+\varepsilon )p} \,\mathrm {d}x\mathrm {d}t\\&\le \tfrac{1}{\varepsilon p} \iint _{Q_{2R}} |F|^{(1+\varepsilon )p} \,\mathrm {d}x\mathrm {d}t. \end{aligned}$$

We insert the preceding estimates above and multiply by $\varepsilon p$. This leads to

$$\begin{aligned} \iint _{{\varvec{E}}_k(R_1,\lambda _1)}&|Du|_k^{(1-\vartheta +\varepsilon )p}|Du|^{\vartheta p} \,\mathrm {d}x\mathrm {d}t\\&\le \lambda _1^{\varepsilon p} \iint _{{\varvec{E}}_k(R_1,\lambda _1)} |Du|_k^{(1-\vartheta )p}|Du|^{\vartheta p} \,\mathrm {d}x\mathrm {d}t\\&\quad + \tfrac{c\, \varepsilon }{1-\vartheta } \iint _{{\varvec{E}}_k(R_2,\lambda _1)} |Du|_k^{(1-\vartheta +\varepsilon )p}|Du|^{\vartheta p}\,\mathrm {d}x\mathrm {d}t+ c\iint _{Q_{2R}} |F|^{(1+\varepsilon )p} \,\mathrm {d}x\mathrm {d}t.\end{aligned}$$

The previous inequality is now combined with the corresponding inequality on the complement $Q_{R_1}\setminus \varvec{E}_k(R_1,\lambda _1)$, i.e. with the inequality

$$\begin{aligned} \iint _{Q_{R_1}\setminus {\mathbf {E}}_k(R_1,\lambda _1)}&|Du|_k^{(1-\vartheta +\varepsilon )p}|Du|^{\vartheta p} \,\mathrm {d}x\mathrm {d}t\\&\le \lambda _1^{\varepsilon p} \iint _{Q_{R_1}\setminus {\mathbf {E}}_k(R_1,\lambda _1)} |Du|_k^{(1-\vartheta )p}|Du|^{\vartheta p} \,\mathrm {d}x\mathrm {d}t. \end{aligned}$$

Taking into account that $|Du|_k\le |Du|$, the result is the estimate

$$\begin{aligned} \iint _{Q_{R_1}} |Du|_k^{(1-\vartheta +\varepsilon )p}|Du|^{\vartheta p} \,\mathrm {d}x\mathrm {d}t&\le \frac{c_*\varepsilon }{1-\vartheta } \iint _{Q_{R_2}} |Du|_k^{(1-\vartheta +\varepsilon )p}|Du|^{\vartheta p} \,\mathrm {d}x\mathrm {d}t\\&+ \lambda _1^{\varepsilon p} \iint _{Q_{2R}} |Du|^{p} \,\mathrm {d}x\mathrm {d}t+ c\iint _{Q_{2R}} |F|^{(1+\varepsilon )p} \,\mathrm {d}x\mathrm {d}t, \end{aligned}$$

where $c_*=c_*(n,p,q,\nu ,L)\ge 1$. Now, we choose

$$\begin{aligned} 0 < \varepsilon \le \varepsilon _o {:}{=} \frac{1-\vartheta }{2c_*}. \end{aligned}$$

Note that $\varepsilon _o$ depends only on $n,p,q,\nu$, and *L*. Furthermore, $\lambda _1^\varepsilon \equiv (\eta B\lambda _o)^\varepsilon \le B \lambda _o^\varepsilon$, since $B\ge 1$, $\eta <1$, and $\varepsilon \le 1$. Moreover, we recall the definition of *B* in (6.15). Therefore, the last inequality implies that for each pair of radii $R_1$, $R_2$ with $R\le R_1<R_2\le 2R$ the following estimate holds:

$$\begin{aligned} \iint _{Q_{R_1}}&|Du|_k^{(1-\vartheta +\varepsilon )p}|Du|^{\vartheta p} \,\mathrm {d}x\mathrm {d}t\\&\le \tfrac{1}{2} \iint _{Q_{R_2}} |Du|_k^{(1-\vartheta +\varepsilon )p}|Du|^{\vartheta p} \,\mathrm {d}x\mathrm {d}t\\&\quad + c\bigg (\frac{2R}{R_2-R_1}\bigg )^{\frac{dp^\sharp (n+2)}{p^\sharp +q-1}} \lambda _o^{\varepsilon p} \iint _{Q_{2R}} |Du|^{p} \,\mathrm {d}x\mathrm {d}t+ c\iint _{Q_{2R}} |F|^{(1+\varepsilon )p} \,\mathrm {d}x\mathrm {d}t. \end{aligned}$$

Now, we apply the Iteration Lemma 2.1 to the last inequality and arrive at the estimate

$$\begin{aligned} \iint _{Q_{R}}&|Du|_k^{(1-\vartheta +\varepsilon )p}|Du|^{\vartheta p} \,\mathrm {d}x\mathrm {d}t\\&\le c\lambda _o^{\varepsilon p} \iint _{Q_{2R}} |Du|^{p} \,\mathrm {d}x\mathrm {d}t+ c\iint _{Q_{2R}} |F|^{(1+\varepsilon )p} \,\mathrm {d}x\mathrm {d}t. \end{aligned}$$

In the left-hand side, we now pass to the limit $k\rightarrow \infty$ with the help of Fatou’s lemma. Subsequently we take means on both sides. This givesRecalling the definition of $\lambda _o$ from (6.13) the preceding inequality turns intoNote that $c=c(n,p,q,\nu ,L)$. For the estimate of the right-hand side, we use the energy estimate from Lemma 3.1 with $a=0$ and $\lambda =\theta =1$ in the formJoining the two preceding estimates, we arrive atwith $c=c(n,p,q,\nu ,L)$, provided $Q_{8R}\subset \Omega _T$. This implies the asserted quantitative estimate (1.7) by a straightforward covering argument. If we choose $\varepsilon \le \varepsilon _1{:}{=}\min \{\varepsilon _o,\frac{\sigma }{p}-1\}$, then the right-hand side is finite, and we deduce $Du\in L_{\mathrm {loc}}^{(1+\varepsilon _1)p}(\Omega _T)$. This completes the proof of Theorem 1.2.

## Data Availability

Data sharing not applicable to this article as no datasets were generated or analysed during the current study.
